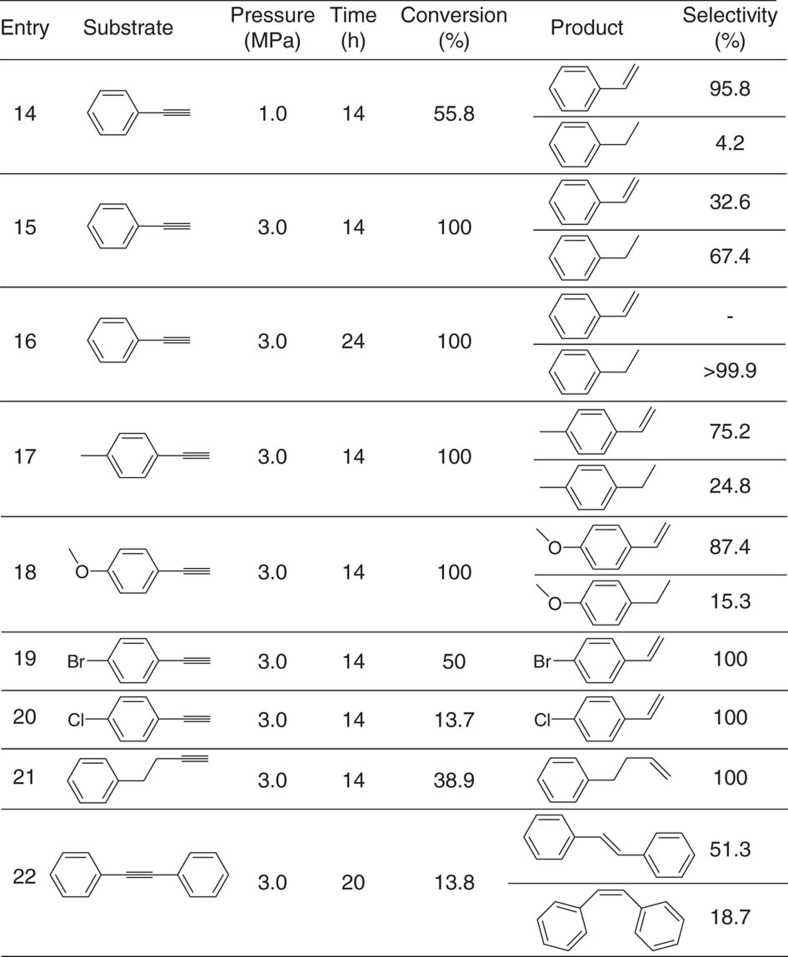# Solid frustrated-Lewis-pair catalysts constructed by regulations on surface defects of porous nanorods of CeO_2_

**DOI:** 10.1038/ncomms15266

**Published:** 2017-05-18

**Authors:** Sai Zhang, Zheng-Qing Huang, Yuanyuan Ma, Wei Gao, Jing Li, Fangxian Cao, Lin Li, Chun-Ran Chang, Yongquan Qu

**Affiliations:** 1 Center for Applied Chemical Research, Frontier Institute of Science and Technology and State Key Laboratory for Mechanical Behavior of Materials, Xi'an Jiaotong University, XiYi Hall, 99 Yanxiang Road, Xi'an, Shannxi 710049, China; 2 Institute of Industrial Catalysis, School of Chemical Engineering and Technology, Xi'an Jiaotong University, Xi'an 710049, China; 3 MOE Key Laboratory for Nonequilibrium Synthesis and Modulation of Condensed Matter, Xi'an Jiaotong University, Xi'an 710049, China; 4 State Key Laboratory of Catalysis, Dalian Institute of Chemical Physics, Chinese Academy of Sciences, Dalian 116023, China

## Abstract

Identification on catalytic sites of heterogeneous catalysts at atomic level is important to understand catalytic mechanism. Surface engineering on defects of metal oxides can construct new active sites and regulate catalytic activity and selectivity. Here we outline the strategy by controlling surface defects of nanoceria to create the solid frustrated Lewis pair (FLP) metal oxide for efficient hydrogenation of alkenes and alkynes. Porous nanorods of ceria (*PN*-CeO_2_) with a high concentration of surface defects construct new Lewis acidic sites by two adjacent surface Ce^3+^. The neighbouring surface lattice oxygen as Lewis base and constructed Lewis acid create solid FLP site due to the rigid lattice of ceria, which can easily dissociate H–H bond with low activation energy of 0.17 eV.

## Abstract (web summary)

Surface engineering of catalysts allows the tailoring of active sites. Here the authors produce a heterogeneous nanoceria catalyst with engineered defects producing active solid frustrated Lewis pair sites, and use these materials for the hydrogenation of alkynes and alkenes.

Catalytic hydrogenation of unsaturated substrates, a fundamental transformation process, is finding prolific industrial reactions, especially for petrochemistry^1^,^2^,^3^. Hydrogen activation by either transition metal complexes or metal-based heterogeneous catalysts have been well developed and successfully applied for various reactions^4^. Recently, frustrated Lewis pairs (FLPs), Lewis acids and bases that are sterically prevented from interaction to form Lewis acid–base adjuncts, can efficiently and cooperatively activate many small molecules (for example, H_2_, CO_2_, and NO) and even strong C–H bond for important catalytic reactions including hydrogenation, hydroamination and carbon dioxide reduction^5^,^6^,^7^,^8^,^9^,^10^,^11^,^12^,^13^,^14^,^15^,^16^,^17^,^18^,^19^,^20^,^21^,^22^,^23^,^24^,^25^.

However, molecular-based homogeneous FLP catalytic systems raise the difficulties in product purification and catalyst recovery. Therefore, the development of heterogeneous catalysts with FLP-like activity is extremely expected. Meanwhile, the combination of Au powder as Lewis acid and molecular Lewis bases (for example, imine and nitrile) successfully constructs the first example of semi-heterogeneous FLP catalytic system^26^. In this catalytic system, large quantity of Lewis acids and bases were required to avoid the formation of stable Lewis acid–base complexes. The semi-heterogeneous molecular sieves/B(C_6_F_5_)_3_ FLPs deliver remarkable catalytic activity for hydrogenation of ketones and aldehydes^27^. Graphene synthesized by chemical exfoliation has been reported as efficient catalysts for hydrogen activation through an FLP-like behaviour^28^. When the Lewis acid and base sites are in an adequate distance, the activation of hydrogen becomes possible^29^,^30^,^31^. However, they require restricted demanding reaction conditions with long reaction time and a high H_2_ pressure of 30 bar^28^. Formation of an FLP of the Na^+^H^−^/hydroxyl proton O(H^+^) has been successfully demonstrated for Pt_*x*_/NaY zeolites by using *in situ* neutron diffraction and spectroscopic measurements^32^. Inspired by the catalytic mechanism of molecular FLP catalysts, it is also possible to develop novel heterogeneous FLP-like catalysts if independent Lewis acidic and basic species coexist in one material. Recently, Ozin and colleagues^33^,^34^,^35^,^36^,^37^ has reported that the surface FLP sites of In_2_O_3-*x*_(OH)_*y*_ created by a Lewis acidic coordinately unsaturated surface indium site proximal to an oxygen vacancy and a Lewis basic surface hydroxide site showed the catalytic activity for the CO_2_ reduction by H_2_ in both experimental results and theoretical predictions. It provides a possible strategy to construct solid FLP sites in oxides by creating oxygen vacancy.

Nanostructured ceria is characterized with a large number of surface-bound defects that are primarily oxygen vacancy or Ce^3+^ species as active sites for heterogeneous catalysis^38^,^39^,^40^,^41^,^42^,^43^. In previous investigations, the catalytic ability of ceria is driven by the ability to switch between different oxidation states (Ce^3+^/Ce^4+^ redox pair) accompanied with the storage and release of oxygen when the temperature is higher than 150 °C (ref. 44). At low temperature, the surface oxygen is less mobile and can be firmly held in the lattice matrix^45^,^46^. With these precedents in mind, the combination of the ‘fixed' surface oxygen as Lewis base and surface defects as Lewis acid in ceria is similar to the sterically encumbered pairs of Lewis acids and bases for molecular FLP catalysts, if the surface Lewis acid and base are independent.

We choose ceria as another candidate to demonstrate the possibility to construct FLP sites due to its reversible Ce^3+^/Ce^4+^ redox pair and rich surface chemistry^38^. Ceria has been reported to exhibit the catalytic activity for the selective semi-hydrogenation of alkynes through the H_2_ activation by homolytic or heterolytic pathway and subsequent hydrogenation^47^,^48^,^49^,^50^,^51^. In general, a high temperature is required due to the inherent difficulty of ceria for H_2_ activation. Heterolytic (between lattice Ce^4+^ and O^2−^) pathway is considered as the pathway for H_2_ dissociation due to the relative low activation energy^51^. Hence, it is not surprise to observe the decreased hydrogenation activity of ceria with the increased surface defects^47^,^49^. Incapability for hydrogenation of alkenes and restricted reaction conditions for semi-hydrogenation of alkynes indicate the low hydrogenation activity of ceria.

In this work, solid FLP sites on CeO_2_ surface, which deliver a very high catalytic activity for hydrogenation of alkenes and alkynes with a wide scope under mild conditions (*T*=100 °C and *P*(H_2_)=1.0 MPa), have been successfully created by regulating their surface defects. The richness of surface defects is found to be the key for construction of a new surface Lewis acidic center by two adjacent reduced surface Ce atoms near an oxygen vacancy. The ‘fixed' surface lattice oxygen as Lewis base and constructed Lewis acid have a large possibility to be close enough but independent due to the richness of surface defects and unique geometrical and electronic configurations, similar to the molecular-based FLPs. Porous nanorods of ceria (*PN*-CeO_2_) with a large surface Ce^3+^ fraction of 30.8% associated with a high concentration of oxygen vacancy have been demonstrated as the solid metal oxide FLP catalyst with a low activation barrier of 0.17 eV for H_2_ dissociation.

## Results

### Design strategy of solid FLP catalysts

**Figure 1:**
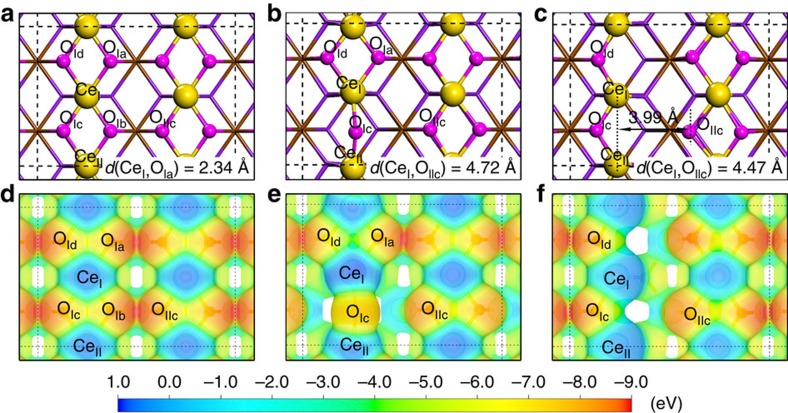
Schematic images of concept for design of solid frustrated Lewis pairs in CeO_2_ crystal structure. (**a**) Optimized structure of ideal CeO_2_(110). (**b**) Optimized structure of CeO_2_(110) with one oxygen vacancy. (**c**) Optimized structure of CeO_2_(110) with two adjacent oxygen vacancies. (**d**) Electron-density isosurface of ideal CeO_2_(110). (**e**) Electron-density isosurface of CeO_2_(110) with one oxygen vacancy. (**f**) Electron-density isosurface of CeO_2_(110) with two oxygen vacancies. The electron-density isosurfaces are plotted at 0.01 *e* bohr^−3^. The colour bar represents the electrostatic potential scale.

Metal oxides possess the surface Lewis acid (metal cation) and Lewis base (oxygen anion), which form the Lewis acid-base adjuncts and deliver no FLP-like activity^51^,^52^. Fortunately, the surface defects induced by the reducible/oxidable valence states of metal cations associated with oxygen vacancy in metal oxides can create novel surface Lewis acidic sites and/or Lewis basic sites. The ‘fixed' surface lattice atoms near the constructed surface defects in a close proximity may be unbonded, which can avoid the formation of classic Lewis acid–base adjuncts due to the rigid crystal lattice. Such a configuration may activate the small inert molecules such as H_2_ and CO_2_. Our hypothesis here is that the activation of inert molecules can be realized by rationally designed surface engineering and regulation on the structural defects of metal oxides to create novel FLP active sites. This possibility is illustrated by taking CeO_2_ as an example in view of both the geometrical and electronic configurations (Fig. 1).

Starting from the ideal CeO_2_(110) facet, the surface Ce and O form Lewis acid–base adjuncts (Fig. 1a). Two kinds of surface configurations, Ce_I_-O_I(a∼d)_ and Ce_I_-O_IIc_, are identified on the ideal CeO_2_(110) surface. Ce_I_ and O_I(a∼d)_ are adjacent and contiguous to each other, forming a classic Lewis acid–base adjunct, whereas the unbonded Ce_I_ and O_IIc_ show a distance of 4.60 Å, which may deliver FLP-like activity. However, the electronic interactions between Ce_I_ and its neighbouring O_Ib_ will block the function of Ce_I_-O_IIc_ pairs as shown in Fig. 1d. Therefore, the removal of oxygen atom directly linked with Ce_I_ is the prerequisite to construct a pair of unbonded Lewis acid and base sites. When the O_Ib_ atom is removed, two surface Ce atoms (including Ce_I_) are reduced and one oxygen vacancy is produced, as demonstrated by the Bader charge population analysis of surface Ce (Supplementary Table 1). After structural relaxation, the O_Ic_ near the oxygen vacancy immigrates to the middle of the Ce_I_ and Ce_II_, leading to Ce_I_ being surrounded by three adjacent oxygen atoms (Fig. 1b). In this case, three types of configurations can be found including Ce_I_-O_Ia/Id_, Ce_I_-O_Ic_ and Ce_I_-O_IIc_, of which the Ce_I_-O_Ia/Id_ and Ce_I_-O_Ic_ form classic Lewis acid–base adjuncts. The Ce_I_-O_IIc_ with a distance of 4.72 Å falls in the domain of solid FLPs to activate small molecules but is still hindered by O_Ic/Ia_ due to the electronic interaction between Ce_I_ and O_Ic/Ia_ (Fig. 1e). When the second surface oxygen (O_Ia_) is removed, two oxygen vacancies and four reduced Ce cations (including Ce_I_ and Ce_II_) are generated (Fig. 1c and Supplementary Table 1). The left oxygen O_Ic_ and O_Id_ will not migrate to the middle of two Ce cations. Thus, the reduced Ce cations (Ce_I_ and Ce_II_) and surface lattice oxygen (O_IIc_) are independent Lewis acid and base, indicating that Ce_I_-O_IIc_ and Ce_II_-O_IIc_ may act as the solid FLPs (Fig. 1c,f), similar to molecular FLPs. In addition, a special FLP-like active site of two adjacent reduced surface Ce sites (Ce_I_ and Ce_II_) and lattice O_IIc_ is constructed and denoted as (Ce_I_,Ce_II_)-O_IIc_ hereafter. Compared with Ce_I_-O_IIc_ and Ce_II_-O_IIc_ configurations, (Ce_I_,Ce_II_)-O_IIc_ with a shorter distance (3.99 Å) and stronger charge contraction by two adjacent reduced surface Ce cations is likely to deliver a higher capability to activate small molecules. On the other hand, the larger concentration of oxygen vacancy also increases the electron density of oxygen atoms (Bader charge analysis, Supplementary Table 1), suggesting the higher ability of oxygen to donate electrons. Bearing the above discussion in mind, it is possible to realize all-solid FLP catalysts of CeO_2_ by regulating the surface defects.

Similar to the CeO_2_(110) surface, the solid FLPs can also be constructed by removing surface oxygen atoms of the CeO_2_(100) surface. The surface oxygen atoms locate in the first atomic layer and surface cerium atoms sit in the second atomic layer on the ideal CeO_2_(100) surface (Supplementary Figs 1a and 14b). The Ce_I_-O_Ia_ pair forms a classic Lewis acid–base adjunct with a distance of only 2.19 Å. Although the Ce_I_-O_Ic_ pair has a distance of 4.41 Å, it fails to be a FLP as the blocking of O_Ib_ next to Ce_I_ (Supplementary Fig. 1c). Removal of O_Ib_ can construct a FLP site, (Ce_I_,Ce_II_)-O_Ic_ with a distance of 3.95 Å (Supplementary Fig. 1b,d), similar to the solid FLP catalyst on the reduced CeO_2_(110) surface. However, the construction of surface FLP site cannot be realized by removing surface oxygen atoms on the CeO_2_(111) facet. Similar to the ideal CeO_2_(110) surface, two types of Lewis acid-base pairs, that is, Ce_I_-O_Ic_ and Ce_II_-O_IIc_, are classic Lewis acid-base pairs on the ideal CeO_2_(111) surface. If one O atom (O_Ic_ in Supplementary Fig. 2a) is removed (Supplementary Fig. 2b), the O_IIc_ atom is still surrounded by Ce_II_ and Ce_III_ without the formation of FLP (Supplementary Fig. 2e). Similarly, when two O atoms (O_Ib_ and O_Id_ in Supplementary Fig. 2a) are removed (Supplementary Fig. 2c), O_Ia_ and Ce_I_ also cannot form FLPs owing to the blocking by Ce_II_* and Ce_III_* (Supplementary Fig. 2f). Therefore, the CeO_2_ nanostructures with the mainly exposed (110) and (100) facets may possess high catalytic hydrogenation activity rising from the formation of the solid FLPs.

### Catalytic hydrogenation activity of *PN-*CeO_2_

To explore the possibility of metal oxides as all-solid FLP catalysts, *PN*-CeO_2_ with mainly exposed (110) and (100) facets, is selected as the model catalysts and hydrogenation of styrene is selected as a feature reaction. *PN*-CeO_2_ has been previously reported with a high surface Ce^3+^ fraction (30.8%) and a large concentration of oxygen vacancy^53^,^54^,^55^,^56^. Besides, the surface properties of *PN*-CeO_2_ can be effectively regulated by post-treatment with well preserved structural features, providing a platform to investigate the correlation between the hydrogenation activity of ceria and their surface properties.

**Figure 2:**
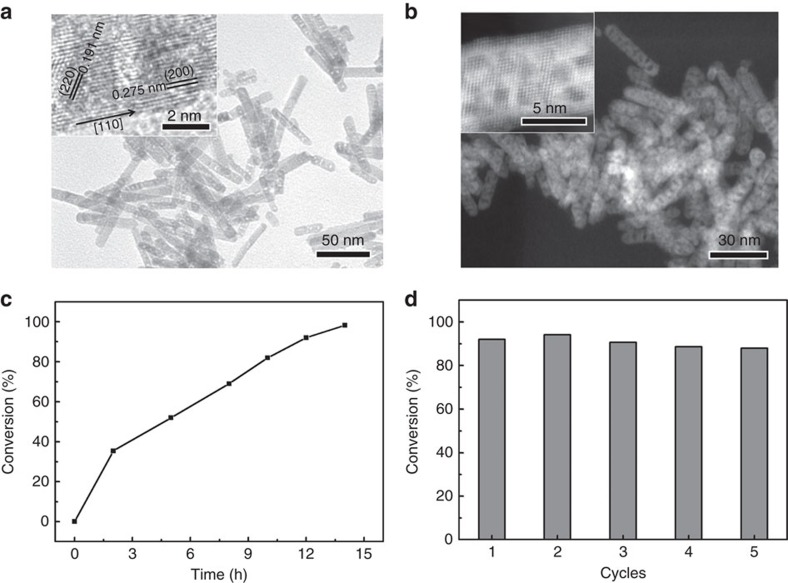
Structural characterization and catalytic performance of *PN*-CeO_2_ catalysts for hydrogenation of styrene. (**a**) Bright-field TEM image of *PN*-CeO_2_. Inset is the high resolution TEM image of *PN*-CeO_2_. (**b**) Dark-field TEM image of *PN*-CeO_2_. High resolution TEM image of inset displays a pore size of 2.94±0.78 nm. (**c**) Time course of styrene conversion catalysed by *PN*-CeO_2_. (**d**) Recyclability of *PN*-CeO_2_ catalysts for hydrogenation of styrene. Reaction conditions: styrene (1.0 mmol), toluene (0.5 ml) and *PN*-CeO_2_ (20.0 mg) at 100 °C and 1.0 MPa H_2_ pressure.

*PN*-CeO_2_ was synthesized through a two-step hydrothermal synthetic approach, in which nonporous nanorods (Supplementary Fig. 3) with the mixed phases of Ce(OH)_3_ and CeO_2_ was obtained in the first-step hydrothermal process at 100 °C and porous nanorods with cubic fluorite structure were synthesized by dehydration/oxidation of precursor nanorods at the second hydrothermal treatment at 160 °C. Both bright-field and dark-field transmission electron microscopic (TEM) studies reveal a porous rod-like morphology with a dimension of∼8 × 60 nm (Fig. 2a,b). X-ray diffraction (XRD) analysis confirms the precursor nanorods undergo the dehydration of the Ce(OH)_3_ content being transformed into CeO_2_ at 160 °C (Supplementary Figs 3b and 4). High-resolution TEM (inset Fig. 2a) image of *PN*-CeO_2_ exhibits two kinds of lattice fringes of (220) and (200), which have respective inter-planar spacings of 0.191 and 0.275 nm. Similar to previous reports, the nanorods preferentially grow along [110] direction and are enclosed by (220) and (200) planes^57^. Derived from dark-field TEM images, the average pore size is 2.94±0.78 nm, which is similar as the average value of 2.2 nm obtained from the gas adsorption isotherms (Supplementary Fig. 5a,c,d). Surface area of *PN*-CeO_2_ determined by Brunauer–Emmett–Teller analysis is 122 m^2^ g^−1^ (Supplementary Fig. 5b).

Initially, optimization of the reaction conditions for hydrogenation reaction catalysed by *PN-*CeO_2_ is shown in Supplementary Table 2. Despite the catalytic activity of *PN-*CeO_2_ had been observed at a low temperature of 60 °C and low H_2_ pressure of 0.2 MPa, an unsatisfied styrene conversion of 34.9% was delivered after 24 h reaction. Raising reaction temperature (100 °C) and H_2_ pressure (1.0 MPa) realized the complete hydrogenation of styrene in 14 h successfully. Figure 2c shows time course of hydrogenation of styrene catalysed by *PN-*CeO_2_. The reaction proceeded continuously to reach 98.2% conversion of styrene in 14 h and afford >99.9% chemoselectivity towards ethylbenzene.

After catalytic hydrogenation reaction, *PN-*CeO_2_ were recovered by centrifugation and washed with ethanol for three times. Before reuse, the spent *PN-*CeO_2_ catalysts were treated at 220 °C for 4 h under the argon protection. Afterwards, *PN-*CeO_2_ can achieve five-time recyclability without any obvious catalytic degradation (Fig. 2d). The unaltered morphology, unchanged phase and almost no changed surface Ce^3+^ fraction of the spent *PN*-CeO_2_ illustrate their structural and catalytic stability during hydrogenation reactions (Supplementary Fig. 6).

### Catalytic screening of various metal oxides

**Figure 3:**
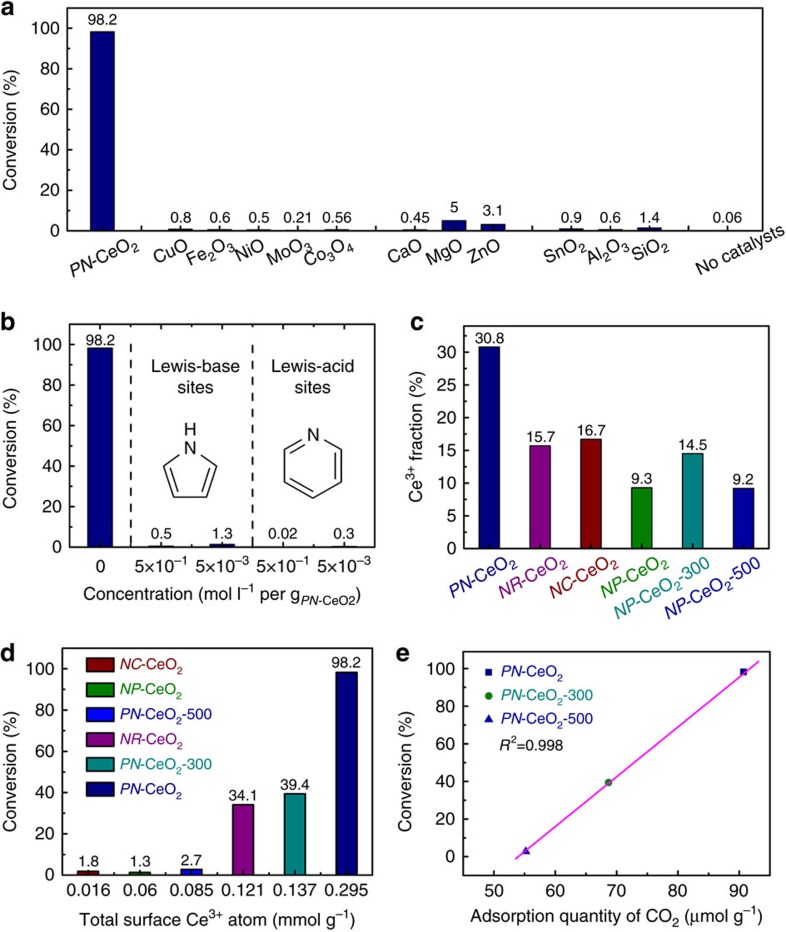
Mechanism investigations on hydrogenation activity of *PN*-CeO_2_. (**a**) Catalytic activity of various metal oxides for hydrogenation of styrene. (**b**) Influences of molecular Lewis-base or Lewis-acid on the catalytic activity of *PN*-CeO_2_ for hydrogenation of styrene. (**c**) Catalytic activity of nanoceria for hydrogenation of styrene. (**d**) Effects of the total surface Ce^3+^ atom number for hydrogenation of styrene. (**e**) Correlation between conversion of styrene and adsorption quantity of CO_2_ on CeO_2_ catalyst. Reaction conditions: styrene (1.0 mmol), toluene (0.5 ml) and catalysts (20.0 mg) at 100 °C and 1.0 MPa H_2_ pressure for 14 h.

Derived from the previous studies on the semi-hydrogenation of alkynes by ceria^47^, *PN-*CeO_2_ with a high concentration of surface defects should inactive or even inert for hydrogenation of styrene. To understand the high catalytic activity of *PN-*CeO_2_ in the current work, the catalytic activity of various metal oxides including acidic oxides, basic oxides and transition metal oxides for hydrogenation of styrene were also investigated under 100 °C and 1.0 MPa for 14 h. As presented in Fig. 3a, the typical acidic oxides (SiO_2_, Al_2_O_3_ and SnO_2_) delivered a very poor catalytic activity with styrene conversions below 1.4%. Poor catalytic activity of basic oxides (CaO, MgO and ZnO) was also observed. Meanwhile, the styrene conversions obtained from other common transition metal oxides, CuO, Fe_2_O_3_, NiO, MoO_3_ and Co_3_O_4_, were below 0.8%, indicating their inertness for the hydrogenation of alkenes under the operated conditions.

Control experiments (Fig. 3a) indicated that the hydrogenation reaction of styrene could not happen in the absence of *PN*-CeO_2_, indicating the nature catalytic ability of *PN*-CeO_2_. The major difference between *PN*-CeO_2_ and other metal oxides is the coexistence of Lewis acidic and basic sites in high concentrations for *PN*-CeO_2_. Therefore, the survey of metal oxides for hydrogenation of styrene indicates that the absence of either acidic or basic sites on the surface of metal oxides results in near complete deactivation for the hydrogenation of alkenes.

To further confirm the necessity of the co-existence of Lewis acidic and basic sites of *PN*-CeO_2_ in current catalytic system, hydrogenation of styrene was performed in the presence of other molecular Lewis acid or base during the catalytic process. The Lewis base pyridine and Lewis acid pyrrole as common molecules are generally used to confirm the presence of Lewis acidic and basic sites for metal oxides, respectively. As a strong base pyridine (strong acid pyrrole) can be able to absorb on the Lewis acidic (Lewis basic) sites of *PN*-CeO_2_ immediately, when they are added to the reaction solution. Therefore, the FLPs can be effectively blocked by the pyridine and pyrrole molecule. As shown in Fig. 3b, addition of trace amount of either Lewis base pyridine or Lewis acid pyrrole completely terminated the hydrogenation reactions due to the blockage of surface Lewis acidic or basic sites by those small molecules, respectively, similar to that of molecular FLPs^58^,^59^. Trace amount of pyridine/pyrrole molecules were adsorbed on the surface Lewis acidic/basic sites of CeO_2_, leading to a low possibility for the formation of surface FLP sites in the aspect of population of adjacent surface Ce^3+^. The blockage of either Lewis basic sites or Lewis acidic sites of *PN*-CeO_2_ leads to the deactivated hydrogenation of alkenes, revealing that the coexistence of Lewis acidic and basic sites on the surface of *PN*-CeO_2_ are essential to activate H–H bond and achieve high activity in the current system.

### Role of surface defects of *PN*-CeO_2_ for hydrogenation

For the semi-hydrogenation of alkynes by ceria, hydrogen dissociative adsorption on surface oxygen leads to the formation of two surface –OH groups and dissociation of C–H bond of R−C≡C−H on the adjacent surface Ce and O atoms^49^. In those studies, three points were outlined: (1) the obtained alkenes cannot be further reduced into alkanes; (2) experimental results suggest that the hydrogen activation process is the rate-limiting step due to the poor ability of ceria to cleave H−H bond; (3) the catalytic activity of ceria can be significantly decreased in the presence of large amount of surface defects. Followed by the literature reports, *PN*-CeO_2_ with a high surface concentration of oxygen vacancy should deliver a poor catalytic activity for hydrogenation reactions. In fact, *PN*-CeO_2_ indeed shows a very high catalytic activity for styrene hydrogenation, suggesting a different catalytic mechanism for the current work. Besides, both alkenes and alkynes can be reduced by *PN*-CeO_2_ under much mild conditions (see Discussion below), also revealing an alternative catalytic pathway for hydrogen activation and subsequent hydrogenation.

To explore the roles of surface defects of ceria, hydrogenation of styrene was also performed for ceria nanoparticles (*NP*-CeO_2_) with a surface Ce^3+^ fraction of 9.3% (Supplementary Fig. 7), ceria nanocubes (*NC*-CeO_2_) with a surface Ce^3+^ fraction of 16.7% (Supplementary Fig. 8) and non-porous ceria nanorods (*NR*-CeO_2_) with a surface Ce^3+^ fraction of 15.7% (Supplementary Fig. 9). The conversions of styrene were only 1.3%, 1.8% and 34.1% for *NP*-CeO_2_, *NC*-CeO_2_ and *NR*-CeO_2_ under the identical reaction conditions of those catalysed by *PN*-CeO_2_ for 14 h, respectively (Fig. 3d). The surface area of 98 m^2^ g^−1^ for *NR*-CeO_2_ is close to that of *PN*-CeO_2_ of 122 m^2^ g^−1^ (Supplementary Table 3), indicating surface area is not the critical factor to determine their difference in their catalytic activity for hydrogenation of alkenes. The high consistence between the surface Ce^3+^ fractions and the catalytic activity of ceria reveals that the hydrogenation activity of ceria is determined by the abundance of the surface defect sites.

As predicted from the theoretical calculations, it is also possible to create the surface FLP sites on CeO_2_(100) surface. However, the catalytic activity of *NC*-CeO_2_ was much lower than that of *PN*-CeO_2_. Considering the small surface area of *NC*-CeO_2_, we also performed the hydrogenation of styrene by 200 mg *NC*-CeO_2_ with the comparable surface area to that of 20 mg *PN*-CeO_2_ under the identical conditions. A very low styrene conversion of 5.3% was observed, which could be attributed to the low concentration of surface defects of *NC*-CeO_2_ with a Ce^3+^ fraction of 16.7%. The results further confirm that the concentration of surface defects is the main factor to construct large amount of surface FLP sites without regard to the morphology of CeO_2_ catalysts.

Hydrogenation performance of *NR*-CeO_2_ (20 mg) and *NC*-CeO_2_ (200 mg) catalysts suggested that the catalytic activity of FLP sites on CeO_2_(110) was higher than that of CeO_2_(100) with similar surface Ce^3+^ fractions (Fig. 3c,d). It can be attributed to the different steric configuration of FLP sites on CeO_2_(110) and CeO_2_(100). Both the acidic and basic sites of the constructed FLP sites on CeO_2_(110) are in the top atomic layer (Fig. 1c,f and Supplementary Fig. 14a), whereas the basic sites (O atoms) on CeO_2_(100) are in the top atomic layer with the second atomic layer of the acidic sites (Ce atoms) (Supplementary Figs 1d and 14b). Both two configurations can effectively activate hydrogen molecule. However, styrene can encounter less steric hindrance when reacting with the intermediate of heterolysis of H_2_ at the FLP sites on CeO_2_(110). Besides, due to the high surface energy, oxygen atoms of CeO_2_(100) is more prone to diffuse on surface than that of CeO_2_(110)^60^. This diffuse of oxygen atoms (basic sites) can break the steric encumbered structures of FLP sites, leading to the decrease in activity of FLP sites or even the formation of classic acid-base adduct. Thus, the FLP sites on CeO_2_(100) are less stable than those on CeO_2_(110), indicating that the stability of FLP sites could be another factor in the decrease of activity on CeO_2_(100) surface.

The surface Ce^3+^ fractions of *PN*-CeO_2_ were effectively reduced from 30.8% of as-synthesized catalysts to 14.5 and 9.2% (Fig. 3c and Supplementary Fig. 10), when *PN*-CeO_2_ was treated at 300 °C (obtained *PN*-CeO_2_-300 catalysts) and 500 °C (obtained *PN*-CeO_2_-500 catalysts) in air for 8 h, respectively. The calcinated *PN*-CeO_2_ preserved the porous morphology (Supplementary Fig. 11), as well as their surface areas (Supplementary Table 3). After calcination, the obtained *PN*-CeO_2_ catalysts were immediately used for hydrogenation of styrene under the same conditions. The 39.4% and 2.7% conversions of styrene were yielded with *PN*-CeO_2_-300 and *PN*-CeO_2_-500, respectively. The decreased catalytic activity can only be attributed to their decreased concentration of surface oxygen vacancy due to the high temperature annealing at 300 and 500 °C in air.

On the other hand, the *PN*-CeO_2_ was reduced under 10% H_2_/Ar at 200 °C for 2 h and then transferred from the reducing atmosphere to the hydrogenation system immediately. The exposure of the reduced *PN*-CeO_2_ to air was controlled 5 min to minimize the re-oxidation of the reduced catalysts. The catalytic activity for reduced *PN*-CeO_2_ also enhanced from 69.1% to 86.4% after 8 h reaction (Supplementary Fig. 12). The enhanced catalytic activity can be attributed to the increased concentration of surface oxygen vacancy, leading to an increased possibility to construct more surface FLP sites. Therefore, the parallel experiments further strengthen the evidence linking the concentration of surface oxygen vacancy of *PN*-CeO_2_ with their catalytic activity for hydrogenation of alkenes.

The decreased hydrogenation activity of *PN*-CeO_2_-300 and *PN*-CeO_2_-500 can be further revealed from the temperature-programmed hydrogen desorption (H_2_-TPD) tests. All *PN*-CeO_2_ catalysts were exposed to H_2_ at 50 °C and the weakly physisorbed hydrogen was removed by flushing Ar for 20 min. A desorption peak at 190 °C was observed for all *PN*-CeO_2_ catalysts (Supplementary Fig. 13), indicating a strong interaction between H_2_ and *PN*-CeO_2_. With the increase of calcination temperature, the decreased peak intensities demonstrated the reduced amount of activated hydrogen on *PN*-CeO_2_-300 and *PN*-CeO_2_-500. Therefore, the decreased catalytic activity of the calcined *PN*-CeO_2_ was delivered for styrene hydrogenation.

### Mechanism investigations

All control experiments strongly suggest that the coexistence of Lewis acidic and basic sites plays extremely important roles in activating hydrogen at mild conditions and realising their high catalytic activity for hydrogenation reactions. The results are totally different to the catalytic mechanism of the semi-hydrogenation of alkynes by ceria^47^,^48^,^49^. In Fig. 1, we have illustrated that it is possible to create FLP active sites through regulations on the surface defects of CeO_2_(110). In general, the key features of organic molecules FLP catalysts are defined as the independent Lewis acid and base in the presence of sterically substituents on Lewis acid and base to preclude the formation of the classical Lewis acid–base adducts^30^,^61^. Addition of any other small Lewis acid or base molecules results in the formation of adducts and completely blocks the catalytic activity of FLP catalysts during the catalytic process^9^,^12^,^28^. On the basis of the known essentiality of molecular FLP catalysts and a strong correlation between the hydrogenation activity and surface properties of *PN*-CeO_2_ with coexistence of Lewis acid and base in current system, it's logical to rationalize the *PN*-CeO_2_ as all-solid FLP catalysts. The analogous aspects of molecular FLP catalysts and *PN*-CeO_2_ are listed: (1) independent surface Lewis acidic and basic sites of *PN*-CeO_2_ (Fig. 1c); (2) close proximity (4∼5 Å) between surface Lewis acidic site and basic site for activation or splitting of small molecules; and (3) completely quenched hydrogenation activity with addition of other Lewis acids or bases.

**Figure 4:**
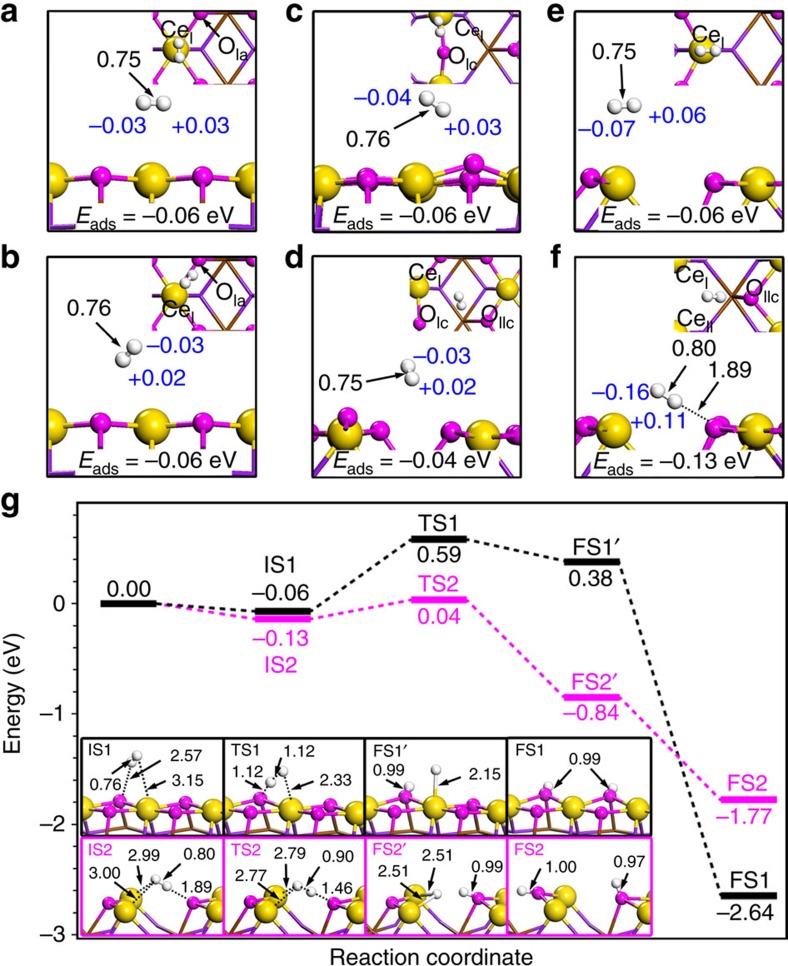
The H_2_ adsorption and dissociation pathways on the surface of *PN*-CeO_2_. The optimized structures of H_2_ adsorption on (**a**) Ce_I_ atom of ideal CeO_2_(110); (**b**) Ce_I_-O_Ia_ pair of ideal CeO_2_(110); (**c**) Ce_I_-O_Ic_ pair of CeO_2_(110) with one oxygen vacancy; (**d**) Ce_I_-O_IIc_ pair of CeO_2_(110) with one oxygen vacancy; (**e**) Ce_I_ atom of CeO_2_(110) with two oxygen vacancies; and (**f**) (Ce_I_,Ce_II_)-O_IIc_ pair of CeO_2_(110) with two oxygen vacancies in both top (inset) and side views. The adsorption energies (*E*_ads_), the bond distances (in Å) colored in black, and the Bader charge population (in *e*) colored in blue are also shown. (**g**) Energy profiles for H_2_ dissociation on ideal CeO_2_(110) in black curve and CeO_2_(110) with two oxygen vacancies in red curves. The optimized structures of initial states (IS), transition states (TS) and final states (FS) are labeled with bond distance (in Å). The zero energy reference corresponds for the sum energy of H_2_ in the gas phase and the corresponding clean CeO_2_ surfaces.

To support the aforementioned conclusions, DFT calculations using the Vienna *Ab-initio* Simulation Package^62^,^63^,^64^ were performed to investigate the adsorption and activation of hydrogen on the CeO_2_(110) surface. For ideal CeO_2_(110) (Supplementary Fig. 14a), hydrogen molecule prefers to be adsorbed at the top site of Ce_I_ (Fig. 4a) or classic acid–base site (Fig. 4b) with weak adsorption energies of −0.06 eV, in consistence with previous studies^65^,^66^. The H–H bond length of the weakly adsorbed H_2_ is 0.75 and 0.76 Å for the two adsorption configurations, respectively. These values are close to that of gas phase H_2_ (0.75 Å), suggesting that H_2_ is difficult to be activated on ideal CeO_2_(110). The Bader charge populations of the physisorbed H_2_ (Fig. 4a,b) further indicate that H_2_ is slightly polarized and hardly activated on ideal CeO_2_(110). With one oxygen vacancy constructed on CeO_2_(110), the adsorption and activation of H_2_ is still poor on both Ce_I_-O_Ic_ (Fig. 4c, −0.06 eV) and Ce_I_-O_IIc_ sites (Fig. 4d, −0.04 eV), which is further evidenced by the long distance (∼3.0 Å) between H_2_ and CeO_2_(110) surface and the similar H−H bond length of adsorbed H_2_ to that of gas-phase H_2_. For the surface with two adjacent oxygen vacancies, H_2_ preferentially adsorbs at the created solid FLP site (Ce_I_,Ce_II_)-O_IIc_ (Fig. 4f) rather than on the top site of the unsaturated Ce atom (Fig. 4e). The results reveal the easy activation of H_2_ molecule at the site of (Ce_I_,Ce_II_)-O_IIc_: (1) a relatively larger adsorption energy of H_2_ of −0.13 eV (Fig. 4f). (2) A significantly elongated H−H bond length of 0.80 Å. (3) A shorter distance between H_2_ and the CeO_2_(110) than those in other adsorption configurations (Fig. 4a-e). The distance between one hydrogen atom of H_2_ and the base site of solid FLPs is only 1.89 Å. (4) A larger polarization of H−H bond due to the electrostatic interactions between the FLPs and H_2_, as indicated in the Bader charge of −0.16 *e* and +0.11 *e* for two hydrogen atoms (Fig. 4f). The aforementioned aspects strongly suggest that the novel solid FLP site constructed by two Ce cations and one O anion is more effective in H_2_ adsorption and activation than the classic acid–base adjuncts.

To further confirm the high activity of such a constructed solid FLP site towards the activation of small molecules, the dissociative activation of H_2_ is investigated on the ideal CeO_2_(110) and reduced CeO_2_(110) surface in the presence of (Ce_I_,Ce_II_)-O_IIc_. From the energy profile depicted by Fig. 4g (black curve), the dissociation of H_2_ on ideal CeO_2_(110) starts from a physisorbed state (IS1) and surpasses an activation barrier of 0.65 eV to reach a dissociated state (FS1'). In FS1', the dissociated hydrogen bound to Ce site is meta-stable and readily diffuses to nearby surface oxygen, leading to the formation of a more stable FS1. The H_2_ dissociation on CeO_2_(110) with the solid FLP active sites experiences a similar process, which also starts from a physisorbed state. Noticeably, the dissociation barrier is only 0.17 eV on the constructed FLP site, which is 0.48 eV lower than that on ideal CeO_2_(110). Moreover, the dissociation of H_2_ on solid FLPs (from IS2 to FS2') is exothermic by 0.71 eV in contrast to the endothermic energy (0.44 eV, from IS1 to FS1') on ideal CeO_2_(110). Both kinetic and thermodynamic results suggest that the dissociative activation of H_2_ is more favourable on the reduced CeO_2_(110) with active FLP sites. The dissociated hydrogen anchored at the Ce site (FS2') can immigrate to an adjacent oxygen to form FS2 with an exothermic energy of 0.93 eV. It is worth noting that FS2 is less stable than FS1, despite two hydrogen atoms are both adsorbed at surface oxygen in two configurations. The calculations suggest that the O–H bond in FS2 is weaker than that in FS1. The weakness of O–H bond is attributed to the enhanced basicity of oxygen on the reduced CeO_2_(110), which is detrimental to the adsorption of H according to the rules of Lewis acid-base pairs on oxide surfaces^67^. Fortunately, the less stable O–H bonds are expected to benefit the subsequent hydrogenation reactions.

Derived from DFT calculations, CeO_2_ nanorods with a high concentration of surface oxygen defects deliver a high possibility to create surface (Ce_I_,Ce_II_)-O_IIc_ FLP sites, yield an enhanced capability of dissociation of H_2_ and improve their catalytic activity for hydrogenation of styrene. The calculation results (Fig. 4) are highly consistent with experiments (Fig. 3), supporting the solid FLP-like activity of *PN*-CeO_2_ regulated and constructed by the surface oxygen defects as illustrated in Fig. 1. To further confirm this conclusion, the correlation between catalytic activity and the concentration of the surface oxygen vacancy of CeO_2_ were studied through the TPD of CO_2_ on the various *PN*-CeO_2_-*T* catalysts. The annealed *PN*-CeO_2_ at 300 and 500 °C exhibited the identical morphological features to those of as-synthesized *PN*-CeO_2_ (Supplementary Fig. 11). Importantly, the *PN*-CeO_2_-300 (120 m^2^ g^−1^) and *PN*-CeO_2_-500 (117 m^2^ g^−1^) showed similar surface areas to that of as-synthesized *PN*-CeO_2_ (122 m^2^ g^−1^). Thus, such a system provides a straightforward way to understand the correlations between the acidity/basicity of *PN*-CeO_2_ and their catalytic activity.

In previous reports, CO_2_ as a Lewis acid can bind with O^2−^ surface ions forming carbonate species with various anchoring structures (bridged, bidentate, monodentate and polydentate)^50^. Furthermore, the presence of oxygen vacancy on CeO_2_ surface can make a strong interaction with CO_2_ and thus enhance its surface basicity^58^. The surface adsorption quantity of CO_2_ is the highest on *PN*-CeO_2_ (90.7 μmol g^−1^), followed by *PN*-CeO_2_-300 (68.7 μmol g^−1^), and the least on *PN*-CeO_2_-500 (55.2 μmol g^−1^) from the CO_2_-TPD results (Supplementary Fig. 15 and Supplementary Table 4). The reduced surface basic sites mainly derived from the obviously decreased medium and strong basic sites, which indicated the decreased amount of surface oxygen vacancy. Meanwhile, the conversions of styrene showed a near linear decrease with the decreased adsorption quantity of CO_2_ on various *PN*-CeO_2_ catalysts (Fig. 3e, *R*^2^=0.998). Results further demonstrate the importance of the surface abundance of defects in CeO_2_ catalysts to construct the surface FLP sites once more.

The correlation between the concentration of oxygen vacancy and their catalytic activity is further investigated with Raman spectra analysis. The concentration of oxygen vacancy is indexed by the ratio of the integrated peak area under the bands at 460 cm^−1^ (vibrational mode of CeO_2_ fluorite structure) and 600 cm^−1^ (oxygen vacancy) of the Raman spectra of *PN*-CeO_2_ annealed at various temperatures (Supplementary Fig. 16)^28^. The decrease in the A_600_/A_460_ with the increased calcination temperature indicates a decrease in the number of oxygen vacancy, as well as the surface Ce^3+^ fraction. Thus, high temperature treatment on *PN*-CeO_2_ leads to the decrease in the number of FLPs sites. Therefore, it is not surprising to observe an obviously decreased conversion of styrene with *PN*-CeO_2_-300 and *PN*-CeO_2_-500; even they have almost the same surface areas (Supplementary Table 3).

The conversion of styrene as a function of total surface Ce^3+^ species (Fig. 3d) and the concentration of oxygen vacancy (Fig. 3e and Supplementary Fig. 16b) indicate that the abundance of surface defects is important for the construction of FLP active sites on *PN*-CeO_2_. The high concentration of surface defects introduces a high possibility to form the surface FLP sites as shown in Fig. 1c. Both theoretical calculations and experimental results illustrate that the FLP active sites in *PN*-CeO_2_ are created by regulations on their surface properties.

### Hydrogenation scope of alkenes

The scope of hydrogenation reactions catalysed by *PN*-CeO_2_ was demonstrated in Table 1. When the electron-donating groups of methyl (–CH_3_), tertiary butyl (–C(CH_3_)_3_) and methoxy (–OCH_3_) were introduced in styrene, the reactant conversions of 99.1, 98.1 and 99% were yielded under 0.6, 0.6 and 1.0 MPa H_2_ pressure, respectively (Entry 1, 2 and 3). However, the catalytic hydrogenation activity of *PN*-CeO_2_ was obviously decreased in the presence of electron-drawing groups in the substrates. The introduction of –Br and –Cl afforded the conversions of 98.5% and 79.5% under 1.0 MPa H_2_ pressure for 20 h, respectively (Entry 4 and 5). It can be attributed to the decreased electronic density of vinyl group in the presence of electron-drawing groups in benzene ring. The hydrogenation of cyclooctene into cyclooctane reached a conversion of 90.5% under 1.0 MPa H_2_ pressure for 20 h (Entry 6). When the H at *α* or *β* site of vinyl is replace by other groups, the catalytic activity of *PN*-CeO_2_ for hydrogenation of vinyl is also decreased due to steric hindrance. Compared to 56.3% conversion of α-methylstyrene under 1.0 MPa H_2_ for 20 h (Entry 7), *trans*-stibene and *cis*-stilbene only reached 10.3% and 8.9% conversions under the same reaction conditions, respectively (Entry 8 and 9).

In light of both experimental and theoretical investigations, the introduction of oxygen vacancy is very important in the formation of FLP active sites for *PN*-CeO_2_. When alkenes include oxygen-containing groups, the blockage of the surface FLP active sites on *PN*-CeO_2_ may happen due to the strong adsorption of oxygen at the surface defects. Thus, the catalytic activity of *PN*-CeO_2_ for those molecules can be reduced or completely depressed. As shown in Table 1 (Entry 10 and 11), *trans*-cinnamaldehyde and allylacetone in the presence of formyl or ketone groups only yielded very low reactant conversions of 21.7% and 12.8%, respectively, despite a high chemoselectivity for olefine products (>99.9%). Owing to the strong interaction between –OH or –NO_2_ groups and surface defects of *PN*-CeO_2_, the activity of FLPs was shielded with 4-hydroxystyrene and 4-nitrostyrene, affording very low conversions (<6%, Entry 12 and 13). There results further support the solid FLP catalytic behaviour of *PN*-CeO_2_ for hydrogenation reactions.

### Hydrogenation scope of alkynes

Ceria has shown the ability to selectively semi-hydrogenated alkynes into olefins^49^. Herein, hydrogenation of various alkynyl substrates was also evaluated with *PN*-CeO_2_ (Table 2). For hydrogenation of phenylacetylene, the conversion was increased from 55.8 to 100% with the increase of H_2_ pressure from 1.0 to 3.0 MPa within 14 h (Entries 14 and 15). However, the selectivity towards styrene was decreased from 95.8 to 32.6% (Entries 14 and 15). By contrast, the selectivity of ethylbenzene was increased from 4.2 to 67.4% (Entry 14 and 15). Only ethylbenzene was obtained with the extended reaction time (Entry 16). The presence of electron-donating methyl group in phenylacetylene yielded 100% conversion under 3.0 MPa H_2_ pressure but a poor chemoselectivity of 75.2% into 4-methylstyrene (Table 2, Entry 17). For 4-methoxy phenylacetylene, similar catalytic behaviour was observed with a 84.7% selectivity of olefin for the conversion of 100% under 3.0 MPa H_2_ pressure at 100 °C (Entry 18). Introducing the electron-drawing substituents on phenylacetylene such as –Br and –Cl, the catalytic activity was reduced, as the low conversions of 50% and 13.7% were yielded for 4-bromo phenylacetylene and 4-chloro phenylacetylene under a H_2_ pressure of 3.0 MPa and a temperature of 100 °C for 14 h, respectively (Entries 19 and 20). For 4-phenyl-1-butyne, 38.9% conversion and 100% selectivity towards olefin products were achieved under 3.0 MPa H_2_ pressure for 14 h reaction (Entry 21). The catalytic activity of *PN*-CeO_2_ was also decreased when the alkynes in the presence of the steric substituents. Under 3.0 MPa H_2_ pressure, hydrogenation of diphenylacetylene only afforded a 13.8% conversion after 20 h reaction (Entry 22). The products of hydrogenation were a mixture of *trans*-stilbene and *cis*-stilbene with the chemoselectivity of 51.3% and 48.7%, respectively (Entry 23).

In summary, we design all-solid metal oxide FLP catalysts through regulations on the surface properties of ceria. As demonstrated by both experimental and theoretical results, *PN*-CeO_2_ with a high concentration of surface defects exhibits the FLP catalytic behaviour for the efficient hydrogenation of alkenes and alkynes with a wide scope. The high density of surface-bound defects is critical to generate the independent surface Lewis acidic and basic sites, construct the surface FLP pair of (Ce_I_,Ce_II_)-O_IIc_ (Fig. 1c) and improve their capability for H_2_ activation with a low activation barrier of 0.17 eV. The capability to hydrogenate alkynes into alkanes further confirms the high hydrogenation activity of *PN*-CeO_2_. The current progress provides a new concept on the structurally correlated catalytic activity of ceria for hydrogenation reactions, which is important for the modern catalyst design. With the ease of preparation, such *PN*-CeO_2_ as the active components in various hydrogenation reactions may be envisioned and so are their ultimate industrial applications.

## Methods

### Preparation of *PN*-CeO_2_ catalysts

Aqueous solutions of Ce(NO_3_)_3_·6H_2_O (1.736 g in 10 ml of deionized water)^53^ and NaOH (19.2 g in 70 ml of deionized water) were mixed slowly. With continuous stirring, the mixture was aged for 30 min before the reaction continued at 100 °C for 24 h in a temperature-controlled electric oven. The reaction mixture was then cooled naturally to room temperature. The products were collected by centrifugation, thoroughly washed with deionized water, and then air-dried at 60 °C. Hydrothermal treatment of this precursor product at 160 °C for 12 h afforded the formation of *PN*-CeO_2_.

### Characterizations

The catalysts were characterized by powder XRD. The XRD patterns with diffraction intensity versus 2*θ* were recorded in a Shimadzu X-ray diffractometer (Model 6000) using Cu *K*_α_ radiation. TEM studies were conducted on the Hitachi HT-7700 with an accelerating voltage of 120 kV. High-resolution and dark-field TEM images were acquired from the Tecnai G2 F20 S-twin TEM at 200 kV. Surface area was measured by N_2_ physisorption (Micromeritics, ASAP 2020 HD88) based on Brunauer–Emmet–Teller method. X-ray photoelectron spectroscopy were acquired using a Thermo Electron Model K-Alpha with Al *K*_α_ as the excitation source.

### Catalytic hydrogenation reactions

For a typical catalytic reaction, 1.0 mmol of substrate and 20.0 mg of catalysts were mixed in 0.5 ml of toluene. The reactions were performed in the autoclave charged with hydrogen with various pressures at the desired reaction temperature. The products were analysed by gas chromatography–mass spectrometry and gas chromatography with m-xylene as the internal standard.

### Data availability

Data supporting the findings of this study are available within the article (and its Supplementary Information files) and from the corresponding author on reasonable request.

## Additional information

**How to cite this article:** Zhang, S. *et al*. Solid frustrated-Lewis-pair catalysts constructed by regulations on surface defects of porous nanorods of CeO_2_. *Nat. Commun.* **8,** 15266 doi: 10.1038/ncomms15266 (2017).

**Publisher's note**: Springer Nature remains neutral with regard to jurisdictional claims in published maps and institutional affiliations.

## Supplementary Material

Supplementary Informattableion Supplementary figures, supplementary tables, supplementary methods and supplementary references.

## Figures and Tables

**Table 1:**
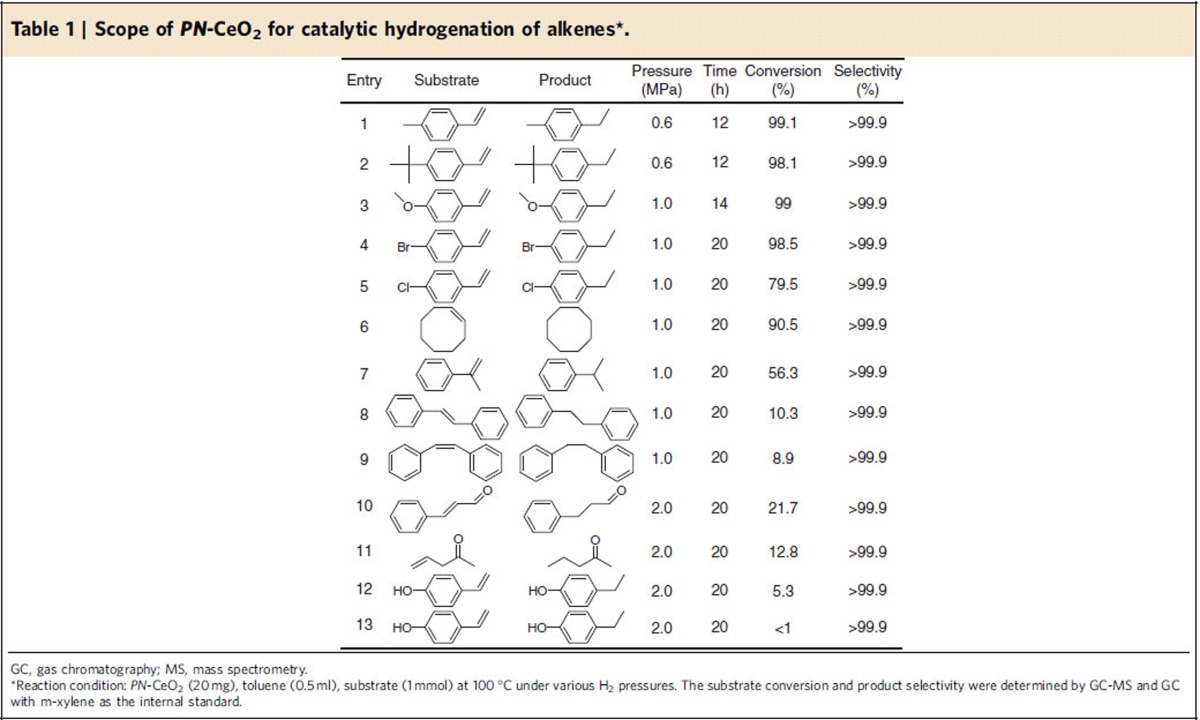
Scope of *PN*-CeO_2_ for catalytic hydrogenation of alkenes^*^.

**Table 2:**
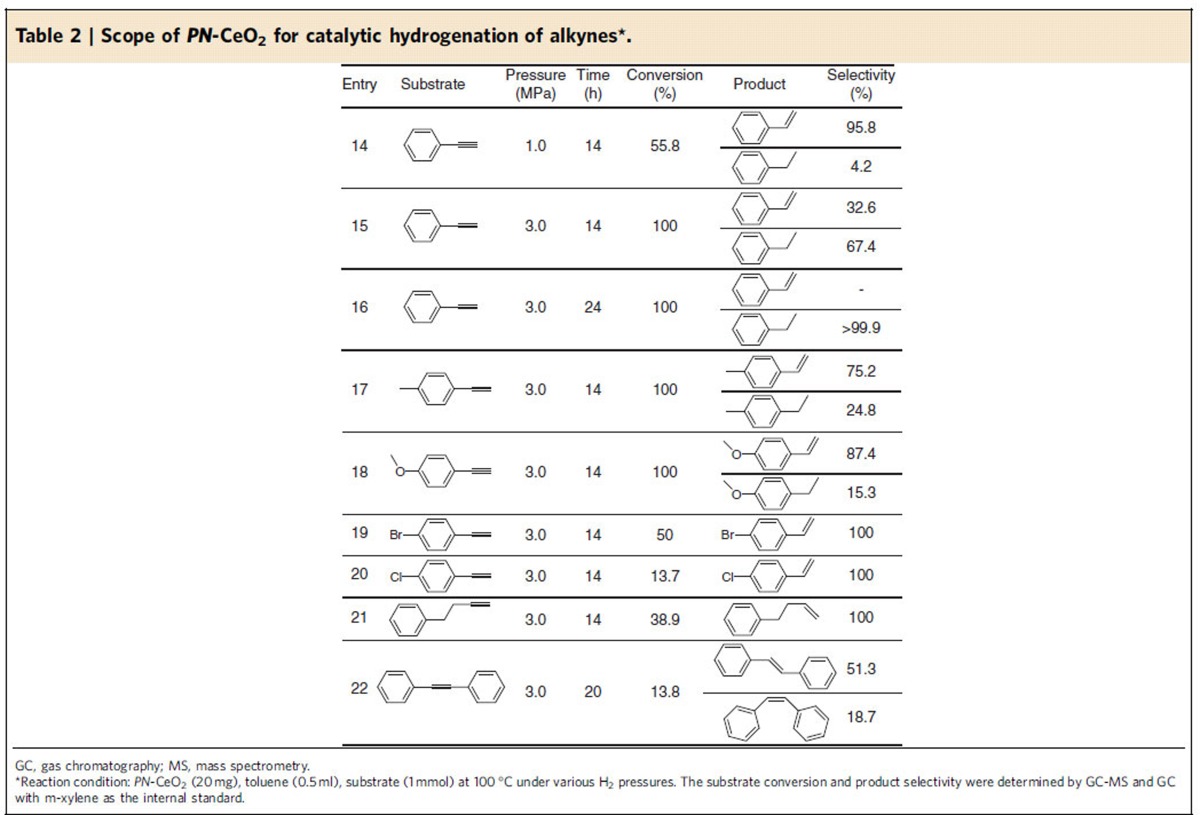
Scope of *PN*-CeO_2_ for catalytic hydrogenation of alkynes^*^.

**Figure:**
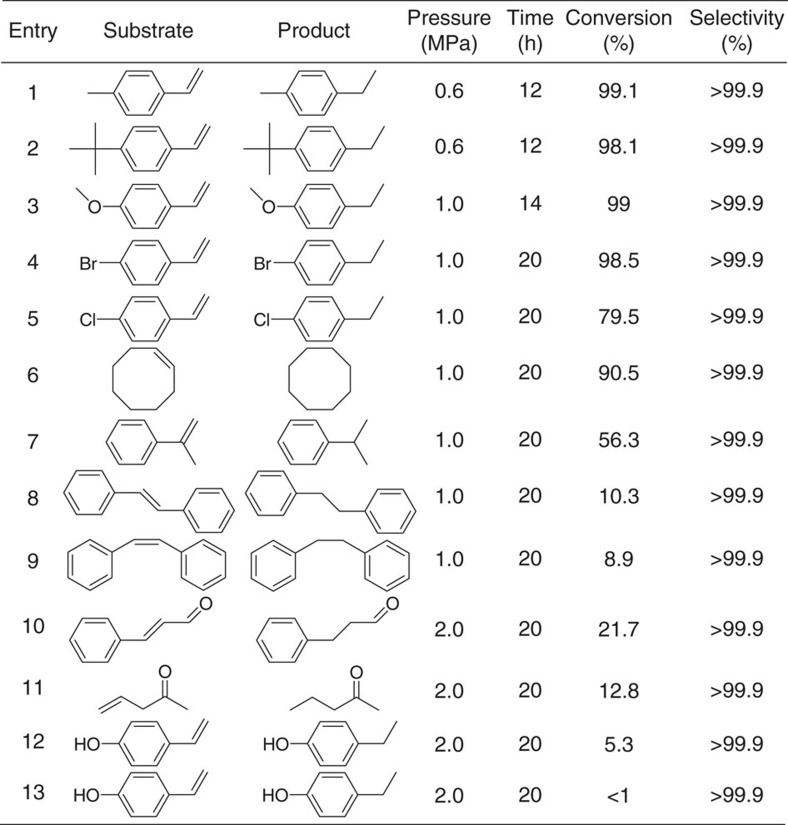


**Figure:**