# Supplementary material for: Solid frustrated-Lewis-pair catalysts constructed by regulations on surface defects of porous nanorods of CeO2
Source: Nat Commun. 2017 May 18;8:15266. doi: 10.1038/ncomms15266 (PMC5454379; doi:10.1038/ncomms15266)
Supplement: Supplementary Informattableion — Supplementary figures, supplementary tables, supplementary methods and supplementary references. [file ncomms15266-s1.pdf]

Supplementary Methods

Synthesis of nanorods CeO_2 (*NR*-CeO_2) catalysts. $Ce(NO_3)_3{\cdot}6H_2O$ (1.736 g) and NaOH (19.2 g) were dissolved in 10 mL and 70 mL of MQ water, respectively. After aging at room temperature for 30 min, the mixture was transferred into a stainless steel autoclave for hydrothermal treatment at 100 ℃ for 24 h. The products were collected by centrifugation, washed with copious amount of water, and dried at 60 ℃.

Synthesis of nanoparticles CeO_2 (*NP*-CeO_2) catalysts. The nanoparticles of CeO_2 were obtained by high temperature calcination of $Ce(NO_3)_3{\cdot}6H_2O$ in air at 500 ℃ for 2 h.

Synthesis of *PN*-CeO_2-300 and *PN*-CeO_2-500 catalysts. The *PN*-CeO_2-300 and *PN*-CeO_2-500 catalysts were prepared by the high temperature annealing of *PN*-CeO_2 in air under 300 ℃ and 500 ℃ for 8 h, respectively.

Synthesis of reduced *PN*-CeO_2 catalysts. The reduced *PN*-CeO_2 was prepared by the high temperature annealing of *PN*-CeO_2 in H_2/Ar 10% under 200 ^oC for 2 h.

Computational Details. DFT calculations were performed using the Vienan *ab* initio Simulation Package (VASP, version 5.3.2).[1-3] The exchange-correction potential function was treated by the generalized gradient approximation (GGA) with the spin-polarized functional of Perdew-Burke-Emzerh (PBE).[4] The wave functions were expanded in a plane wave basis with an energy cutoff of 400 eV, using a projector augmented wave (PAW) method. The DFT + U methodology was used to treat the on-site Coulomb and exchange interactions of the strongly localized Ce 4*f* electrons.[5,6] An effective U = 4.5 eV value was chosen as previous studies suggested.[7] Brillouin zone integration was sampled with the 6 × 6 × 6 and 3 × 3 × 1 Monkhorst-Pack mesh *k*-points for bulk and surface calculations,

respectively.[8] As shown in Supplementary Fig. 14a, the CeO_2(110) surfaces were modeled by a periodic five-layer slab repeated in a 2 × 2 surface unit cell using optimized bulk parameters, a = 5.468 Å, which is in good agreement with experimental value (5.411 Å).[9] The repeated slabs were separated from their neighboring images by a 15 Å vacuum in the direction perpendicular to the surface. The bottom three layers were constrained at the optimized bulk position, and the top two layers with the adsorbates were allowed to relax. The structures were considered relaxed when all forces on each ion were smaller than 0.02 eV/Å, and the convergence criterion for the energy was 10^{-5} eV.

The adsorption energies, E_{ads}, are calculated by using the following equation.

$$E_{\mathrm{ads}} = E_{\mathrm{adsorbate+surface}} - \left(E_{\mathrm{adsorbate}} + E_{\mathrm{surface}}\right)$$

Where $E_{\mathrm{adsorbate+surface}}$ is the total energy of surface covered with adsorbates, $E_{\mathrm{adsorbate}}$ is the energy of adsorbate, and E_{surface} is the energy of clean surface.

To locate the transition state structure of reactions, the minimum-mode following dimer method combined with a Nudged elastic band method was used.[10,11] All transition states were identified by vibration analysis.

CO_2 Temperature-Programmed Desorption (CO_2-TPD) Measurements. CO_2-TPD was carried out using a quartz fixed-bed micro-reactor. For a typical measurement, 150 mg of catalysts were pretreated in Ar at 300 ℃ for 0.5 h. Then, a gas mixture of 10 vol% CO_2/Ar (40 mL min^{-1}) was absorbed by catalysts for 0.5 h at 100 ℃. After the excess CO_2 was removed using Ar (40 mL min^{-1}). The temperature-programmed procedure occurred from 100 ℃ to 800 ℃ with a ramping rate of 10 ℃ min^{-1}. The production of CO_2 was monitored by using a mass spectrometer (QIC-20, HIDEN).

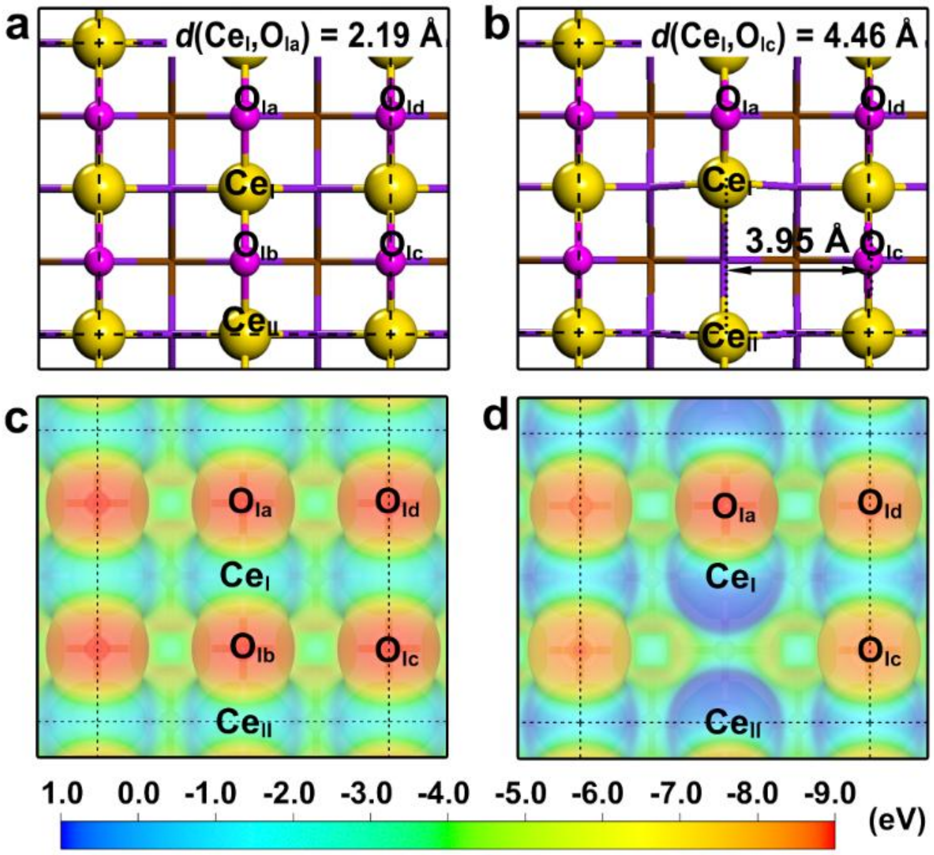

Supplementary Figure 1 | Schematic images for the design of solid frustrated Lewis pairs on CeO_2(100). (a) Optimized structure of stoichiometric CeO_2(100). (b) Optimized structure of CeO_2(100) with one oxygen vacancy. (c) Electron-density isosurface of stoichiometric CeO_2(100). (d) Electron-density isosurface of CeO_2(100) with one oxygen vacancy. The electron-density isosurfaces are plotted at 0.01 *e* $bohr^{-3}$. The color bar represents the electrostatic potential scale.

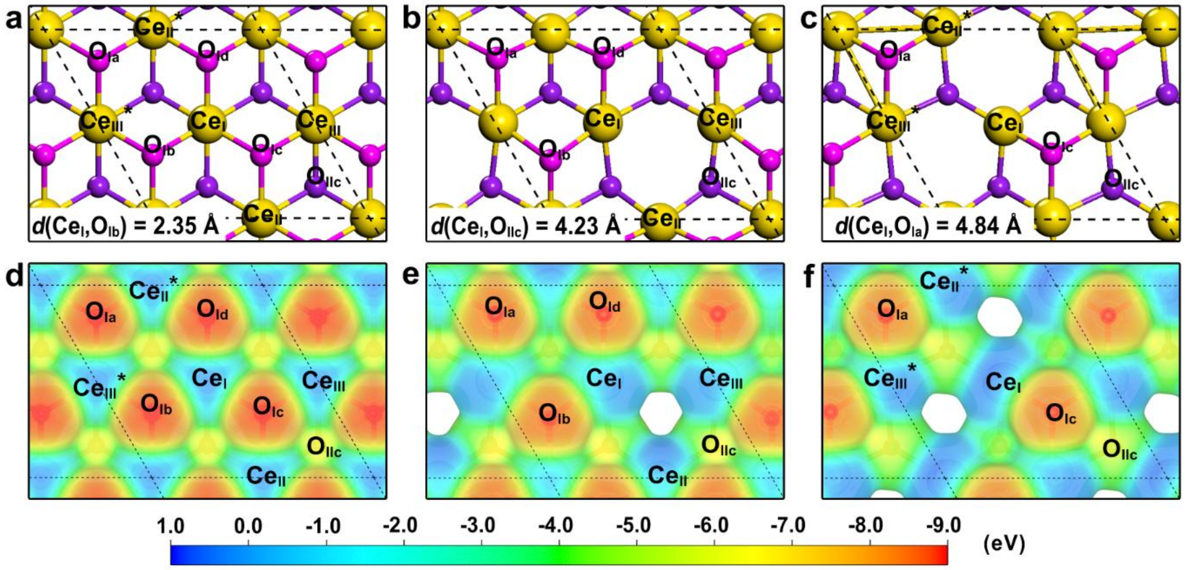

Supplementary Figure 2 | Schematic images for the design of solid frustrated Lewis pairs on CeO_2(111). (a) Optimized structure of ideal CeO_2(111). (b) Optimized structure of CeO_2(111) with one oxygen vacancy. (c) Optimized structure of CeO_2(111) with two oxygen vacancies. (d) Electron-density isosurface of ideal CeO_2(111). (e) Electron-density isosurface of CeO_2(111) with one oxygen vacancy. (f) Electron-density isosurface of CeO_2(111) with two oxygen vacancies. The electron-density isosurfaces are plotted at 0.01 *e* $bohr^{-3}$. The color bar represents the electrostatic potential scale.

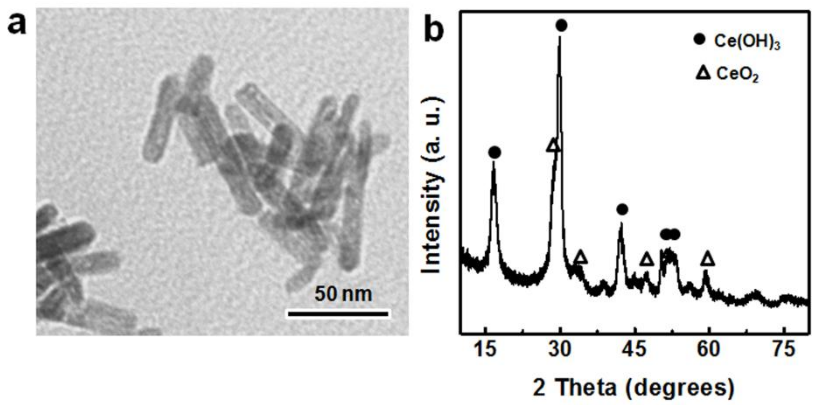

Supplementary Figure 3 | Structural characterization of nonporous precursor nanorods.

(**a**) TEM image.   (**b**) XRD spectrum.

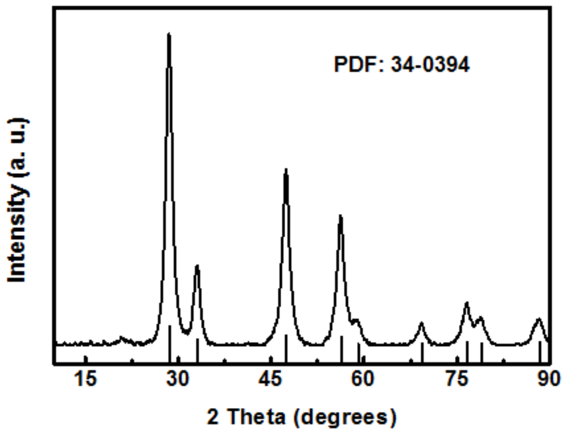

Supplementary Figure 4 | XRD spectrum of *PN*-CeO_2 catalysts.

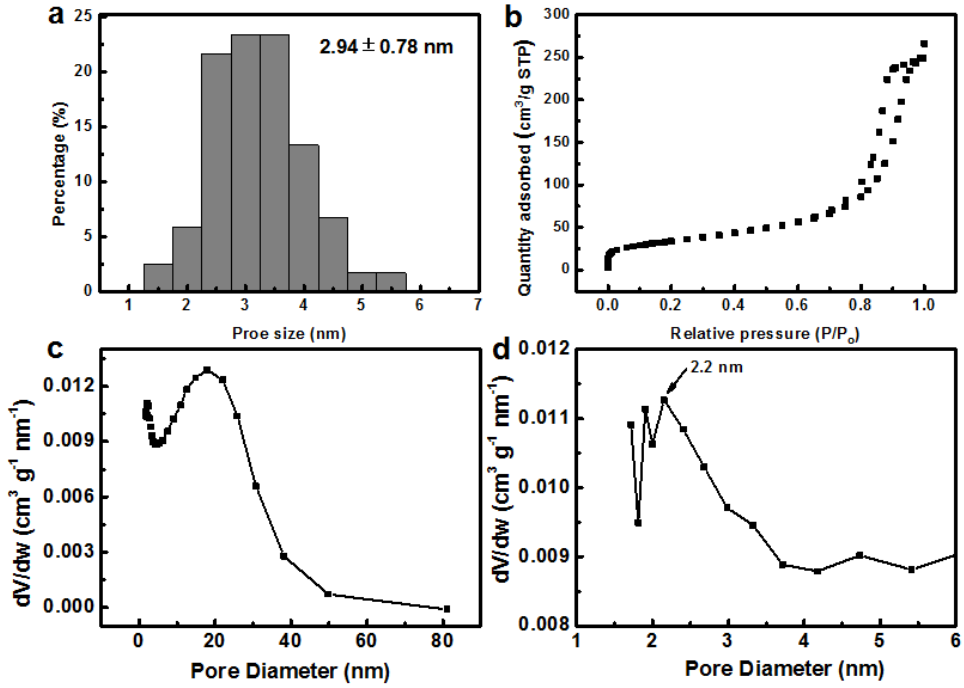

Supplementary Figure 5 | Structural characterization of *PN*-CeO_2 catalysts. (**a**) Pore size distribution of *PN*-CeO_2 catalyst obtained from dark field TEM images. (**b**) Nitrogen adsorption/desorption isotherm plot of *PN*-CeO_2. (**c** and **d**) Pore size distribution of *PN*-CeO_2 catalyst obtained from BET testing.

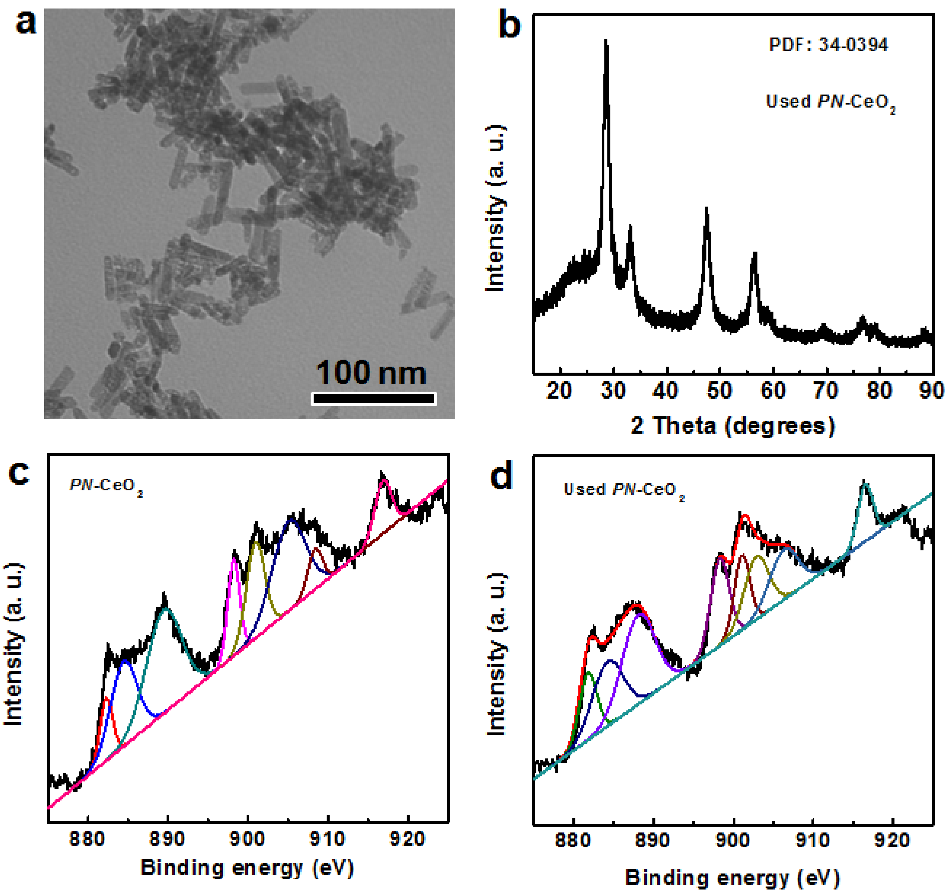

Supplementary Figure 6 | Structural characterization of *PN*-CeO_2 catalysts after 5-cycle hydrogenation of styrene. (**a**) TEM image and (**b**) XRD spectrum of used *PN*-CeO_2; XPS spectrum of (**c**) fresh and (**d**) used *PN*-CeO_2.

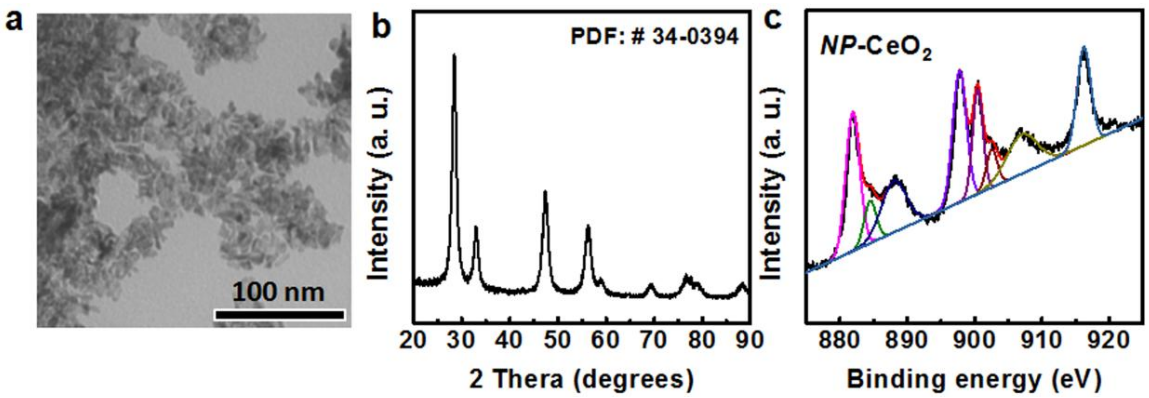

Supplementary Figure 7 | Structural characterization of ceria nanoparticles (*NP*-CeO_2) catalysts. (**a**) TEM image; (**b**) XRD spectrum and (**c**) XPS spectrum.

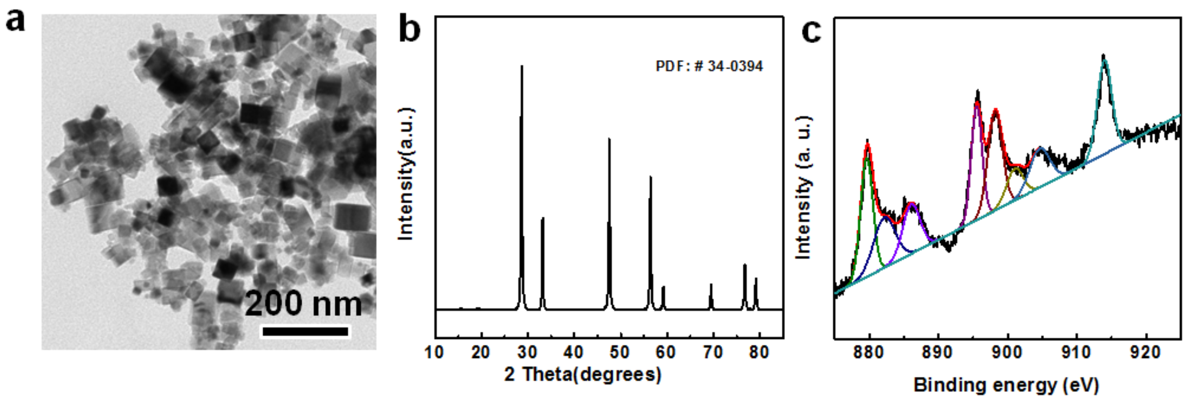

Supplementary Figure 8 | Structural characterization of ceria nanocubes (*NC*-CeO_2) catalysts. (**a**) TEM image; (**b**) XRD spectrum and (**c**) XPS spectrum.

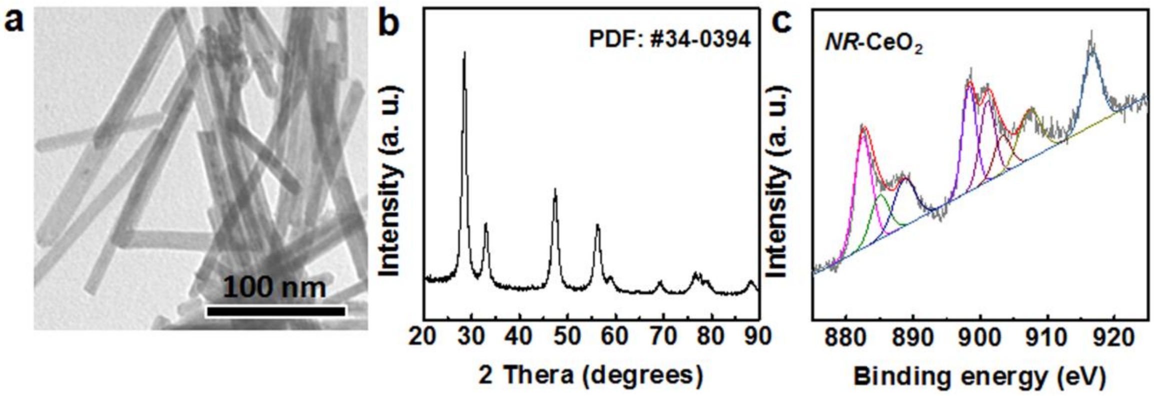

Supplementary Figure 9 | Structural characterization of ceria nanorods (*NR*-CeO_2) catalysts. (**a**) TEM image; (**b**) XRD spectrum and (**c**) XPS spectrum.

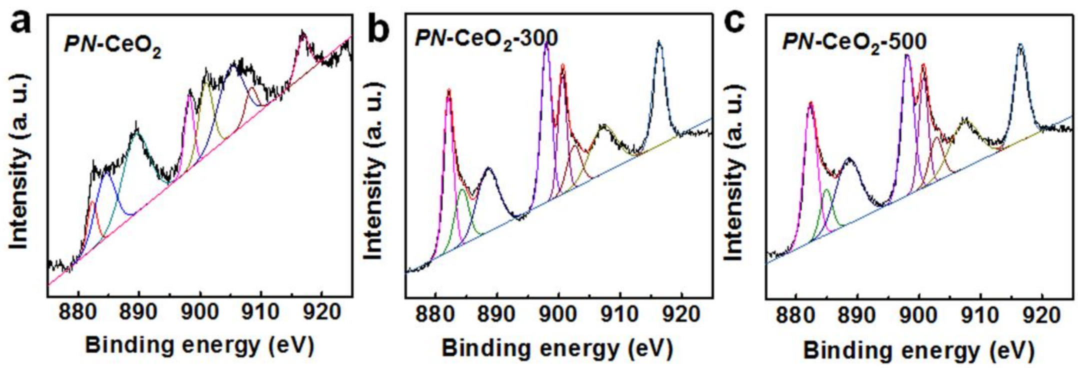

Supplementary Figure 10 | XPS spectra of *PN*-CeO_2 treated at various temperatures. (**a**) As-synthesized *PN*-CeO_2; (**b**) *PN*-CeO_2 treated at 300 ℃; (**c**) *PN*-CeO_2 treated at 500 ℃.

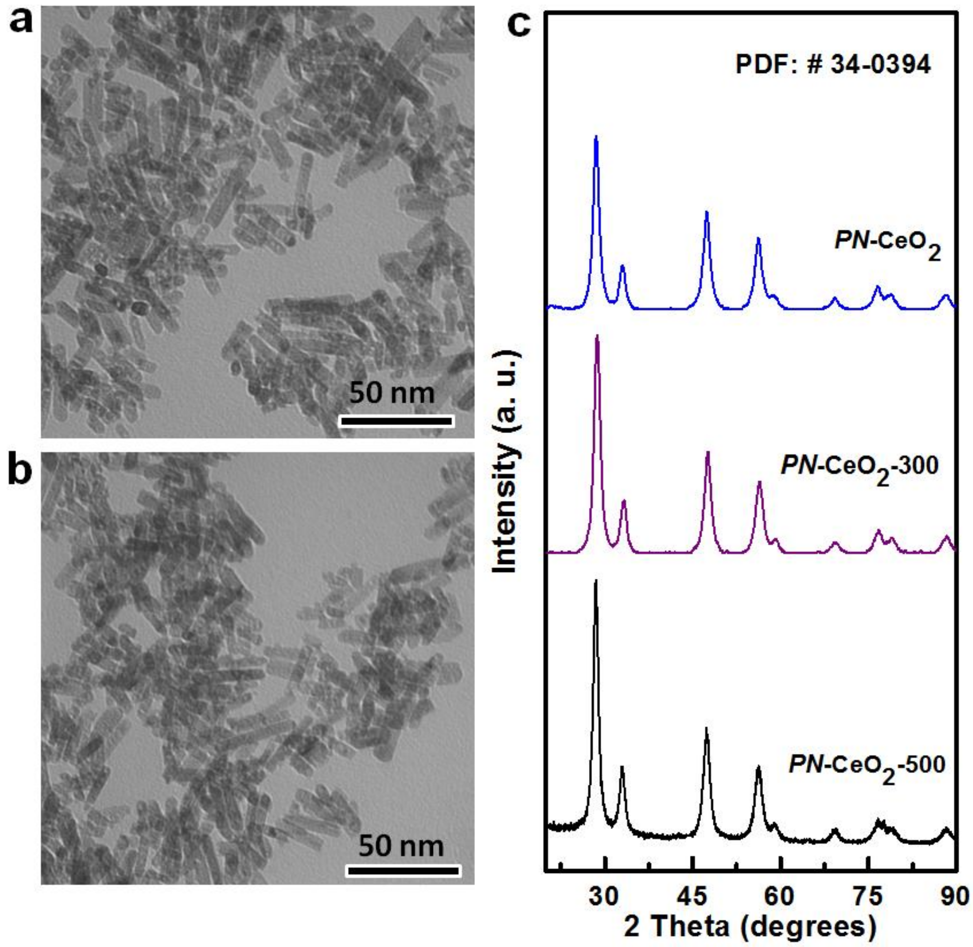

Supplementary Figure 11 | Structural characterization of *PN*-CeO_2 treated at various temperatures. (**a**) TEM images of *PN*-CeO_2 treated at 300 ℃; (**b**) TEM images of *PN*-CeO_2 treated at 500 ℃; (**c**) XRD spectra of the *PN*-CeO_2, *PN*-CeO_2-300 and *PN*-CeO_2-500 catalysts.

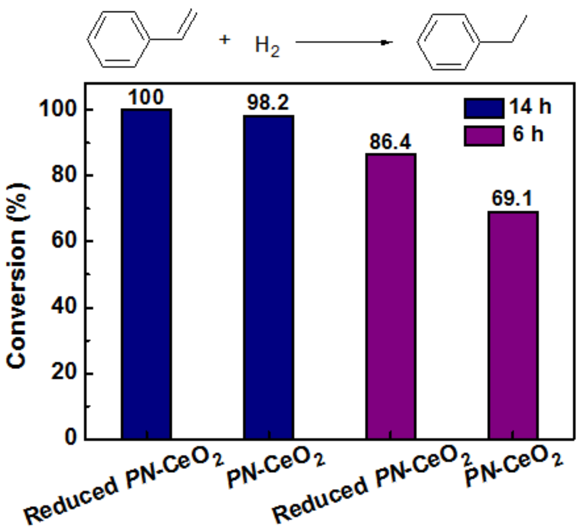

Supplementary Figure 12 | Catalytic activity of *PN*-CeO_2 and reduced *PN*-CeO_2 catalysts for hydrogenation reaction, respectively. Reaction conditions: styrene (1.0 mmol), toluene (0.5 mL), and catalysts (20.0 mg) at 100 ℃ and 1.0 MPa H_2 pressure. For the catalytic reaction by the reduced *PN*-CeO_2, the freshly reduced catalysts were taken out, weighted and transferred into reactor after the naturally cool down under the flow of H_2/Ar. Then, the reactor was purged with H_2 and pumped for three cycles to remove oxygen in the catalytic system. The whole process was controlled about 5 minutes to minimize the re-oxidation of the reduced catalysts. Then, hydrogenation was performed at 100 ℃ and 1.0 MPa H_2 pressure for desired time.

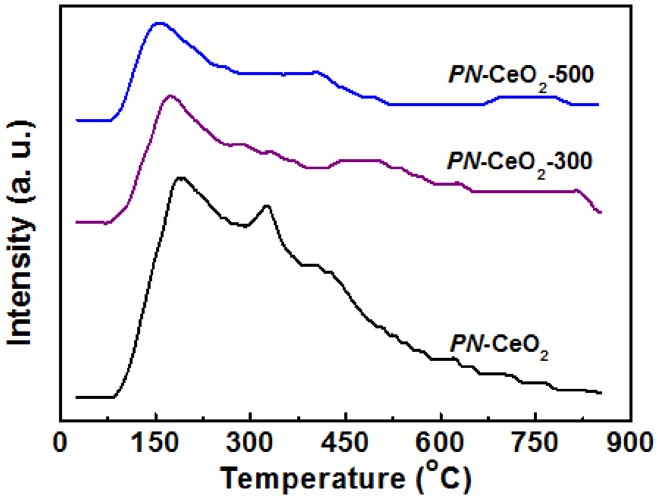

Supplementary Figure 13 | The H_2-TPD desorption profiles after H_2 adsorption at room 50 ^{o}C.

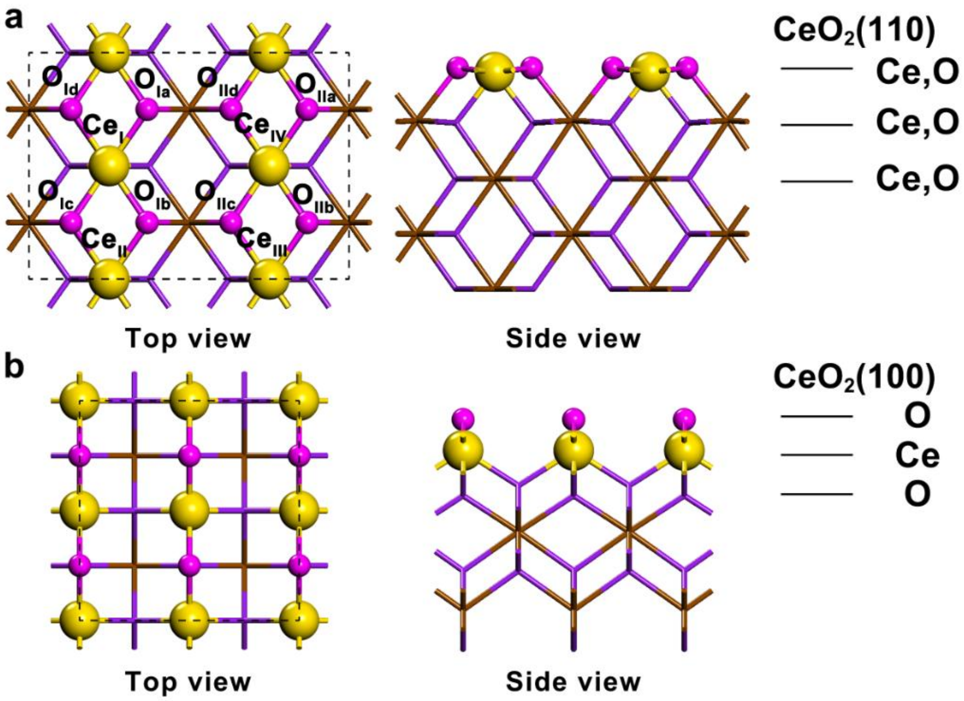

Supplementary Figure 14 | The top and side views of (a) CeO_2 (110) and (b) CeO_2(100), respectively.

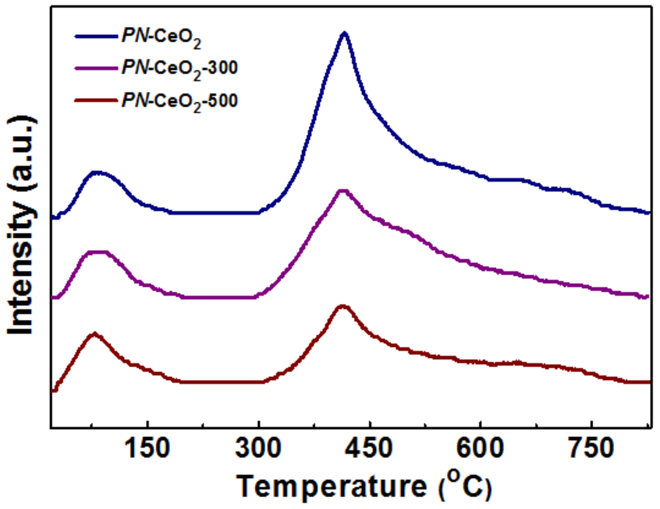

Supplementary Figure 15 | The CO_2-TPD tests for various *PN*-CeO_2-*T* catalysts.

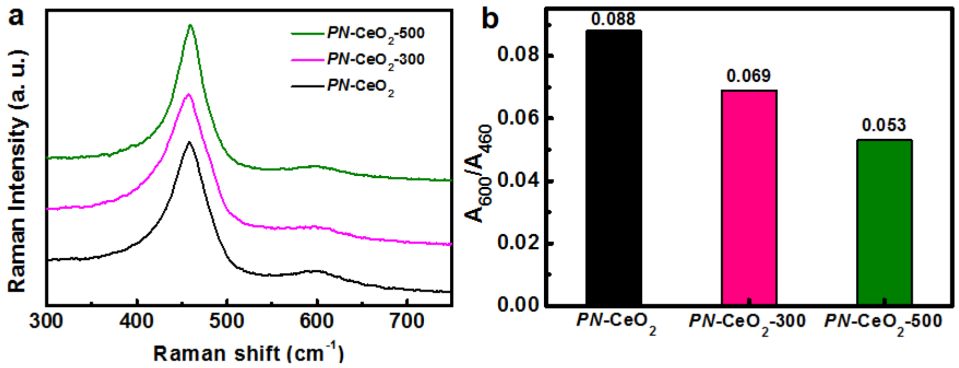

Supplementary Figure 16 | Raman spectra of *PN*-CeO_2 treated at various temperatures.

(**a**) Raman profiles of *PN*-CeO_2 treated at various temperatures (excited by a 514 nm laser).

(**b**) Plot of the ratio of A_{600}/A_{460} of *PN*-CeO_2 calcined at different temperatures in air.

Supplementary Table 1 | Bader charge population of the atoms in the first atomic layer of CeO_2(110)

**Atom**[a]	**Bader charge population / *e***		
	Ideal CeO_2 (110)	CeO_2 (110) with one oxygen vacancy	CeO_2 (110) with two oxygen vacancies
**O_{Ia}**	-1.153	-1.188	/
**O_{Ib}**	-1.163	/	/
**O_{Ic}**	-1.153	-1.230	-1.272
**O_{Id}**	-1.163	-1.204	-1.275
**O_{IIa}**	-1.153	-1.162	-1.212
**O_{IIb}**	-1.153	-1.215	-1.217
**O_{IIc}**	-1.163	-1.227	-1.250
**O_{IId}**	-1.163	-1.179	-1.294
**Ce_{I}**	+2.301	+2.179	+1.931
**Ce_{II}**	+2.301	+2.273	+1.938
**Ce_{III}**	+2.301	+2.320	+2.306
**Ce_{IV}**	+2.301	+2.321	+2.084

[a] The positions of the atoms in the first atomic layer of CeO_2(110) are shown in Supplementary Fig. 14a.

Supplementary Table 2 | Optimization of styrene hydrogenation catalyzed by *PN*-CeO_2

*****

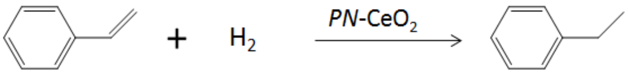

Entry	Temperature (^oC)	Time (h)	Pressure (MPa)	Conversion (%)	Selectivity (%)
1	80	8	0.2	37.1	>99
2	80	8	0.4	59.8	>99
3	80	8	0.6	69.1	>99
4	60	8	0.2	31.8	>99
5	60	24	0.2	34.9	>99
6	80	12	0.2	57.1	>99
7	80	24	0.2	62.5	>99
8	80	18	0.6	82.6	>99
9	100	14	1.0	98.2	>99

* **Reaction condition:** styrene (1 mmol), toluene (0.5 mL) and *PN*-CeO_2 catlaysts (20 mg).

Supplementary Table 3 | Surface properties of various CeO_2 catalysts

Catalysts	BET (m^2/g)	Ce^{3+} fraction (%)	Total surface Ce^{3+} atom (mmol/g)
PN-CeO_2	122	30.8	0.295
NR-CeO_2	98	15.7	0.121
NC-CeO_2	12.2	16.7	0.016
NP-CeO_2	82	9.3	0.06
PN-CeO_2-300	120	14.5	0.137
PN-CeO_2-500	117	9.2	0.085

Supplementary Table 4 | Summary of surface basic sites of various *PN*-CeO_2-*T* catalysts

**Sample**	**Weak (<250 °C)**	**Medium (250-520 °C)**	**Strong (>520 °C)**	**Total (μmol g^{-1})**
PN-CeO_2	10.5	53.8	26.4	90.7
PN-CeO_2-300	10.9	39.0	18.8	68.7
PN-CeO_2-500	12.6	26.2	16.4	55.2

Calculation of the total amount of surface Ce^{3+} atom.

On the basis of 2.117×10^{-19} m^2 area for each surface Ce atom (S) in the (110) crystal face (Supplementary Fig. 17), the amount number of surface Ce^{3+} species on the surface of CeO_2 catalysts can be calculated by the Supplementary Equations (1), (2) and (3):

$$S_{total} = BET * m_{catalysts} \qquad (1)$$

$$N_{Ce} = \frac{S_{total}}{S} \qquad (2)$$

$$N_{Ce^{3+}} = N_{Ce} * f_{Ce^{3+}} \qquad (3)$$

Thus, the amount of surface Ce^{3+} atoms (mmol/g) normalizing at 1.0 g of catalysts can be calculated by the equation (4):

$$n_{Ce^{3+}} = \frac{N_{Ce^{3+}}}{N_A * m_{catalysts}} = \frac{BET * f_{Ce^{3+}}}{S * N_A} \qquad (4)$$

Where $f_{Ce^{3+}}$ is the Ce^{3+} fraction for various CeO_2 catalysts, and N_A is the Avogadro number.

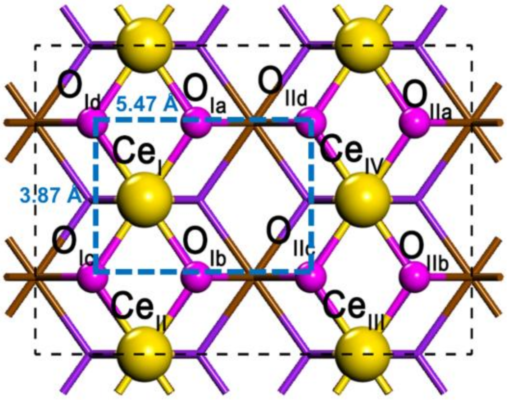

Supplementary Figure 17 | Structure illustration of CeO_2 (110) crystal face.

Supplementary References

1 Kresse, G. & Hafner, J. *Ab initio* molecular-dynamics simulation of the liquid-metal-amorphous-semiconductor transition in germanium. *Phys. Rev. B* **49**, 14251-14269 (1994).

2 Kresse, G. & Furthmüller, J. Efficiency of ab-initio total energy calculations for metals and semiconductors using a plane-wave basis set. *Comp. Mater. Sci.* **6**, 15-50 (1996).

3 Kresse, G. & Furthmüller, J. Efficient iterative schemes for *ab initio* total-energy calculations using a plane-wave basis set. *Phys. Rev. B* **54**, 11169-11186 (1996).

4 Perdew, J. P., Burke, K. & Ernzerhof, M. Generalized gradient approximation made simple. *Phys. Rev. Lett.* **77**, 3865-3868 (1996).

5 Anisimov, V. I., Zaanen, J. & Andersen, O. K. Band theory and Mott insulators: hubbard *U* instead of stoner *I. Phys. Rev. B* **44**, 943-954 (1991).

6 Dudarev, S. L., Botton, G. A., Savrasov, S. Y., Humphreys, C. J. & Sutton, A. P. Electron-energy-loss spectra and the structural stability of nickel oxide: an LSDA+U study. *Phys. Rev. B* **57**, 1505-1509 (1998).

7 Fabris, S., Vicario, G., Balducci, G., de Gironcoli, S. & Baroni, S. Electronic and atomistic structures of clean and reduced ceria surfaces. *J. Phys. Chem. B* **109**, 22860-22867 (2005).

8 Monkhorst, H. J. & Pack, J. D. Special points for Brillouin-zone integrations. *Phys. Rev. B* **13**, 5188-5192 (1976).

9 Kümmerle, E. A. & Heger, G. The structures of C–$Ce_2O_{3+\delta}$, Ce_7O_{12}, and $Ce_{11}O_{20}$. *J. Solid State Chem.* **147**, 485-500 (1999).

10 Henkelman, G. & Jonsson, H. A dimer method for finding saddle points on high dimensional potential surfaces using only first derivatives. *J. Chem. Phys.* **111**, 7010-7022 (1999).

11 Jónsson, H., Mills, G. & Jacobsen, K. W. in *Classical and Quantum Dynamics in Condensed Phase Simulations*. 385-404. (World Scientific Publishing Company, Singapore, 1998).
